# Modeling attention-driven plasticity in auditory cortical receptive fields

**DOI:** 10.3389/fncom.2015.00106

**Published:** 2015-08-19

**Authors:** Michael A. Carlin, Mounya Elhilali

**Affiliations:** Laboratory for Computational Audio Perception, Department of Electrical and Computer Engineering, Johns Hopkins UniversityBaltimore, MD, USA

**Keywords:** auditory attention, feature-based attention, object-based attention, spectro-temporal receptive fields, plasticity, computational model

## Abstract

To navigate complex acoustic environments, listeners adapt neural processes to focus on behaviorally relevant sounds in the acoustic foreground while minimizing the impact of distractors in the background, an ability referred to as top-down selective attention. Particularly striking examples of attention-driven plasticity have been reported in primary auditory cortex via dynamic reshaping of spectro-temporal receptive fields (STRFs). By enhancing the neural response to features of the foreground while suppressing those to the background, STRFs can act as adaptive contrast matched filters that directly contribute to an improved cognitive segregation between behaviorally relevant and irrelevant sounds. In this study, we propose a novel discriminative framework for modeling attention-driven plasticity of STRFs in primary auditory cortex. The model describes a general strategy for cortical plasticity via an optimization that maximizes discriminability between the foreground and distractors while maintaining a degree of stability in the cortical representation. The first instantiation of the model describes a form of feature-based attention and yields STRF adaptation patterns consistent with a contrast matched filter previously reported in neurophysiological studies. An extension of the model captures a form of object-based attention, where top-down signals act on an abstracted representation of the sensory input characterized in the modulation domain. The object-based model makes explicit predictions in line with limited neurophysiological data currently available but can be readily evaluated experimentally. Finally, we draw parallels between the model and anatomical circuits reported to be engaged during active attention. The proposed model strongly suggests an interpretation of attention-driven plasticity as a discriminative adaptation operating at the level of sensory cortex, in line with similar strategies previously described across different sensory modalities.

## 1. Introduction

Plasticity is a ubiquitous property of sensory cortex whereby neural tuning characteristics can be dynamically shaped based on expectations, environmental context, and behavioral demands. Rapid plasticity has been documented across many sensory modalities including vision (Gilbert and Li, 2012), somatosensation (Feldman and Brecht, 2005), olfaction (Mandairon and Linster, 2009), and audition (Schreiner and Polley, 2014). A particularly important driver of neural plasticity is top-down attention, which acts to adapt cognitive resources to selectively focus on behaviorally relevant sensory input. Such a mechanism helps sensory systems dynamically parse the flood of incoming stimuli as environmental context and behavioral demands change over time. For example, attention helps guide the visual search for a friend in a crowd, or it can help a listener follow a specific voice in a cocktail party.

Broadly speaking, attention is a multifaceted and distributed process. Its effects are manifested neurophysiologically at various levels in the cortical hierarchy (Motter, 1993; Fritz et al., 2003; David et al., 2008; Ahveninen et al., 2011; Atiani et al., 2014), cognitively at many levels of abstraction of the raw sensory input (Treisman, 1996; Shinn-Cunningham, 2008), and are dependent on factors such as stimulus statistics (Shuai and Elhilali, 2014), task difficulty (Atiani et al., 2009), and the physical constraints of the underlying neural circuitry (Miller and Buschman, 2013). Nevertheless, a common computational goal can be identified from studies of top-down attention across sensory modalities: that neural tuning characteristics adapt to improve discrimination and separation between the representation of the foreground (i.e., the attended stimuli) and that of the background (i.e., task-irrelevant distractors).

Studies of attention-driven plasticity have a rich history in the visual domain (Itti and Koch, 2001; Itti et al., 2005; Carrasco, 2011). Neurophysiological studies have described a number of neural parameters that are modulated by attention to facilitate foreground/background separation, including response gain (Treue and Trujillo, 1999), feature tuning bandwidth (Spitzer et al., 1988), preferred spatial location (Womelsdorf et al., 2006), and contrast response functions (Martínez-Trujillo and Treue, 2002). Furthermore, these observations can be explained by a plethora of computational models (Frintrop et al., 2010; Borji and Itti, 2013). Early connectionist models describe how attention acts to adapt synaptic weights in a distributed neural network to attend to, and emphasize the representation of, desired spatial locations or features (Olshausen et al., 1993; Tsotsos et al., 1995). More recent efforts have proposed frameworks that unify a variety of attention-driven effects observed in neurophysiological studies, quantifying how attention acts to bias the gains and/or feature tuning functions of neurons to emphasize target-specific features while suppressing the responses to task-irrelevant features (Navalpakkam and Itti, 2007; Reynolds and Heeger, 2009; Borji and Itti, 2014). Overall, these models have been important for establishing a theoretical foundation on which to base questions of the optimal computational strategies for, and the neural substrates of, top-down attention, as well as the meaning, interpretation, and scope of top-down signals (Baluch and Itti, 2011).

In the auditory system, recent neurophysiological studies have begun to shed light on the nature of the computational principles underlying attention-driven plasticity (Fritz et al., 2007a,b; Bajo and King, 2010). Along the central auditory pathway, top-down attentional mechanisms have been shown to dynamically reshape neural tuning characteristics in order to maximize performance of behavioral tasks. These task-driven changes have been summarized by the *contrast filtering hypothesis*, which states that attention acts to enhance representation of attended sounds in the acoustic foreground relative to those in the acoustic background (Fritz et al., 2007c). Particularly striking examples of contrast filtering effects have been observed in primary auditory cortex (A1) via measurements of spectro-temporal receptive fields (STRFs), a kernel often used to summarize the linear processing characteristics of a neuron (Aertsen and Johannesma, 1981; Depireux et al., 2001; Klein et al., 2006). It has been shown that STRFs adapt to directly enhance individual acoustic features of the foreground while suppressing those of the background, and, importantly, that the direction of plasticity reflects the structure of the task and behavioral meaning assigned to foreground and background stimuli (Fritz et al., 2003, 2005c, 2007c; David et al., 2012; Yin et al., 2014). Moreover, despite being subject to dramatic changes in their shape, STRFs exhibit remarkable stability in their tuning characteristics by resisting change over time and/or returning to their nominal shapes post behavior (Elhilali et al., 2007). Furthermore, contrast filtering effects have been observed beyond A1 in secondary auditory belt areas up through executive control areas in prefrontal cortex (Fritz et al., 2010; Atiani et al., 2014). Thus, the computational principles underlying task-driven plasticity can be understood through the lens of a contrast filter that allows the auditory system to dynamically reallocate neural resources in a discriminative fashion to improve performance in specific tasks while maintaining a notion of representational stability over time.

Recent computational modeling efforts have predicted plasticity patterns that are broadly consistent with the contrast filtering hypothesis in A1 (Mesgarani et al., 2010; David et al., 2012). Broadly speaking, these studies propose discriminative cost functions that maximize a notion of distance between neural responses to foreground and background stimuli to determine optimal receptive field parameters subject to biologically plausible constraints. Importantly, these models predict localized differential plasticity effects that reflect the acoustic features of task-relevant stimuli. They are primarily driven by the physical characteristics of the sensory input and represent—by design—models of *feature-based attention*. Although quite informative about computational strategies underlying A1 adaptation patterns, these approaches are limited in two important ways. First, they do not capture the influence of task structure on the direction of plasticity effects. In particular, recent data from mammalian primary auditory cortex suggest that during a tone vs. noise discrimination task, aversive tasks (where the target tone is associated with negative reward) tended to *enhance* representation of the tone whereas appetitive tasks (where the target target is associated with a positive reward) tended to *suppress* representation of the tone (David et al., 2012). Because the models define quadratic cost functions whose optima will not change if the roles of foreground and background are reversed, they are therefore agnostic to task structure, and there is no way to guarantee that plasticity predicted by the models will change direction if the behavioral meanings assigned to foreground and background stimuli are exchanged. Second, because the computational models adapt receptive field parameters based directly on the raw spectro-temporal stimulus—and hence the raw features that characterize the acoustic classes—they lack a mechanism to adapt based on abstractions of the stimulus (e.g., spectro-temporal modulation profile, phase profile, etc.), which one would expect from an *object-based* model of attention that defines the target class along certain characteristics but unconstrains others to allow for variability within the target class.

Inspired by neurophysiological results and previous modeling efforts, this report presents a computational model of attention-driven plasticity of primary auditory STRFs. The model makes explicit two important aspects of top-down attention. The first is that attention defines the acoustic foreground and background by assigning task-relevant categorical labels to observed neural ensemble responses. The second is that attention acts to vary the shapes of STRFs to facilitate improved discrimination between the foreground and background. By designing and optimizing a suitable objective function, we demonstrate that the model predicts STRF changes that are consistent with the contrast filtering hypothesis, in line with those previously observed in physiological studies, and reflect a form of feature-based attention that enhances and suppresses task-salient acoustic cues. Moreover, the form of the model guarantees that the direction of plasticity is consistent with the behavioral meaning of the foreground and background. Next, we explore a generalized form of the discriminative framework that adapts receptive fields based on complex spectro-temporal modulation cues observed in the stimulus, as quantified in the Fourier domain. In this case, the extended model reflects a form of object-based attention, where top-down signals can act on an abstracted representation of the raw acoustic cues. We make predictions for behavioral tasks for which STRF plasticity data is limited or unavailable but could be readily evaluated in neurophysiological studies. Finally, we draw parallels between our model and anatomical circuits thought to be engaged during active attention, and we speculate on the computational goals of these subcircuits.

## 2. Results

### 2.1. Physiological STRF ensemble

**Figure 1:**
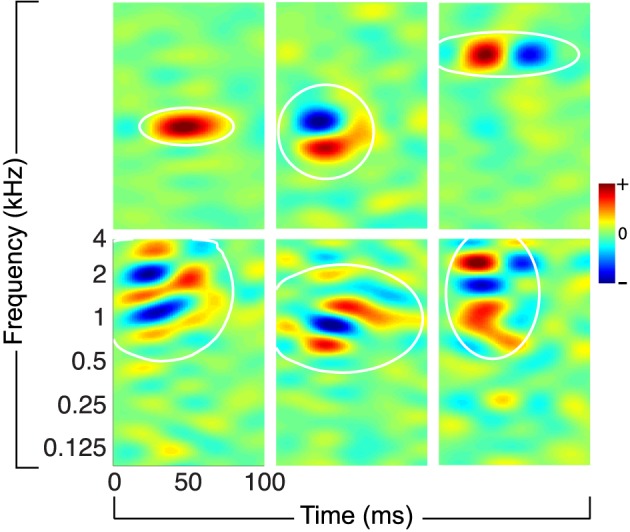
**Examples of physiological STRFs obtained from mammalian primary auditory cortex**. The STRFs reflect sensitivity to a variety of spectro-temporal events in natural sounds, including localized time-frequency energy, spectral and temporal modulations, and more complex joint spectro-temporal modulations. The white ellipses denote isoline contours (at the 20% level) of a localized spectro-temporal mask, defined as a Gaussian envelope fit to each filter (see the main text and the Section 4).

For this study, we consider ensembles of STRFs obtained from recordings of awake, non-behaving ferret primary auditory cortex; some examples from this ensemble are shown in Figure 1. The STRFs reflect sensitivity to a variety of spectro-temporal events that characterize natural sounds, including localized energy in time-frequency, as well as purely spectral, purely temporal, and joint spectro-temporal modulations. For the experiments described below, we consider ten ensembles of *K* = 100 STRFs randomly sampled (with replacement) from a collection of 810 STRFs; more details about ensemble construction are provided in the Section 4.

### 2.2. Overview of the discriminative framework

**Figure 2:**
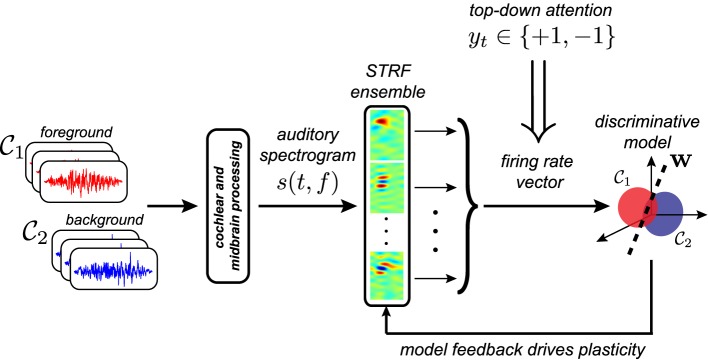
**Proposed discriminative framework for attention-driven plasticity**. Examples of foreground and background stimuli are passed through a model of the auditory periphery, and the resulting auditory spectrogram is analyzed by a bank of STRFs derived from recordings from ferret primary auditory cortex. Top-down attention acts to assign a behaviorally meaningful categorical label to observed population responses, which are subsequently discriminated using logistic regression. Feedback from the discriminative model, in the form of the regressor prediction error, iteratively adapts the shapes of the STRFs to improve prediction of foreground vs. background sounds.

An overview of the discriminative framework considered in this study is shown in Figure 2. Broadly speaking, the proposed framework quantifies the physiologically implied balance between discrimination and stability via an objective function of the form

(1) $$\begin{array}{l} J\left(w,H_{A}\right)=\text{Discriminability}\left(w,H_{A},A_{t},C\right)\\ +\text{Stability}\left(H_{0},H_{A},\lambda \right) \end{array}$$

where **w** is a vector of parameters for a discriminative model, H_0_ and H_*A*_ are the sets of initial and adapted STRFs, respectively, A_*t*_ is a time-varying *attentional signal* that assigns behaviorally meaningful categorial labels to observed neural responses, and (*C*, λ) are hyperparameters that control the impact of each term on the overall objective function. In keeping with nomenclature commonly used in auditory physiological studies, we interchange use of foreground with *target* stimuli, as well as interchange use of background with *reference* stimuli. Thus, the overall goal here is to determine settings of **w** and H_*A*_ that optimize the proposed cost function.

We consider two instantiations of the proposed framework. The *Feature-Based Model* operates directly in the time-frequency domain and operates linearly without constraints on the STRFs. We provide relevant theoretical results and validate the model on behavioral tasks for which physiological results are available, demonstrating that the resulting STRF adaptation patterns directly reflect task-relevant acoustic features. Next, we generalize the framework by considering an *Object-Based Model* that operates on the spectro-temporal modulation profiles of the STRFs with specific constraints on the magnitude and phase of the STRFs. By acting on an abstracted representation of the raw acoustics, this model therefore reflects a form of object-based attention. Again, we present theoretical results for this model. Predictions for behavioral tasks that could be readily evaluated in neurophysiological studies are also provided.

### 2.3. Feature-based model: theoretical results

In the time-frequency domain, we model neural firing rate as

(2) $$\begin{array}{l} r_{k}\left(t\right)=\underset{f}{\sum }\left(m_{k}\left(t,f\right)\cdot h_{k}^{A}\left(t,f\right)\right){*}_{t}s\left(t,f\right) \end{array}$$

where $h_{k}^{A}\left(t,f\right)\in {\mathbb{R}}^{F\times T}$ denotes an STRF we seek to adapt, ^*^_t_ denotes convolution in time, *m*_*k*_(*t, f*) ∈ [0, 1] is a Gaussian-shaped spectro-temporal mask, and *s*(*t, f*) is the stimulus spectrogram. The mask models spectro-temporal constraints related to synaptic input and temporal integration that are typically observed in auditory cortical neurons. Later, we will observe that it guarantees that induced STRF adaptations are also spectro-temporally local. The mask is automatically determined by performing a least-squares fit of a Gaussian envelope to a rectified STRF (see Section 4), and ellipses illustrating the coverage of the masks are shown in Figure 1. Finally, let $r_{t}=\left[1,r_{1}\left(t\right),r_{2}\left(t\right),\cdots ,r_{K}\left(t\right)\right]\in {\mathbb{R}}^{K+1}$ denote an augmented ensemble response.

We model the influence of the top-down attentional signal A_*t*_ as the assignment of a behaviorally relevant categorical label *y*_*t*_ ∈ {+1, −1} to an observed ensemble response **r**_*t*_, where *y*_*t*_ = +1 is associated with a target class of stimuli and *y*_*t*_ = −1 is associated with a reference class. To improve discrimination between target and reference stimuli, we assume that attention acts to vary the shapes of the STRFs in order to maximize the conditional likelihood of the labels. A simple model to quantify this notion is logistic regression, where we model the conditional likelihood as

(3) $$\begin{array}{l} p\left(Y_{t}=y_{t}|r_{t},w\right):=\sigma \left(y_{t}w^{T}r_{t}\right) \end{array}$$

where σ(α) = 1∕(1 + exp(−α))^−1^ is the logistic function and $w=\left[w_{0},w_{1},\cdots ,w_{K}\right]\in {\mathbb{R}}^{K+1}$ is a vector of regression coefficients (Bishop, 2006).

To induce task-driven changes in the STRFs, we define the following objective function:

(4) $$\begin{array}{l} J(w,H_{A})\text{: = }\underset{\text{Discriminability}}{\underset{︸}{\frac{1}{2}||w|{|}_{2}^{2}-C\cdot {\langle \log\sigma \left(y_{t}w^{T}r_{t}\right)\rangle }_{t}}}\\ \text{ + }\underset{\text{Stability}}{\underset{︸}{\frac{\lambda }{2}\sum_{k}||h_{k}^{0}(t,f)-h_{k}^{A}(t,f)|{|}_{F}^{2}}} \end{array}$$

The discriminability terms correspond to the average conditional log-likelihood of the attentional labels with *l*_2_ regularization to prevent the regression coefficients from growing too large and overfitting available training stimuli. The stability term corresponds to an *l*_2_ regularizer on the adapted STRF coefficients that controls “how far” the adapted STRFs can vary from their original versions. This reflects the idea that STRFs resist change and seek to return to their nominal shape upon task completion (Elhilali et al., 2007). Finally, the balance between discriminability vs. stability is controlled by choice of hyperparameters (*C*, λ).

Optimizing *J*(**w**,H_*A*_) is a non-convex problem when trying to *jointly* solve for **w** and H_*A*_, and so there exist many local optima. One strategy for finding these optima is by use of block coordinate descent, where we alternate between two minimization problems:

(P1) $$\begin{array}{ll} \arg\underset{w}{\min}J\left(w,H_{A}\right)\text{ subject to }w_{k}\geq 0, & k=1,2,\cdots \;,K \end{array}$$

(P2) $$\begin{array}{l} \arg\underset{H_{A}}{\min}J\left(w,H_{A}\right) \end{array}$$

We will show below that non-negativity constraints on the regression coefficients are necessary for encoding task valence during adaptation.

Because *J*(**w**,H_*A*_) is a sum of convex functions, and the constraints on (P1) are convex, each subproblem is therefore convex with a unique minimum. Furthermore, since each update to **w** and H_*A*_ does not increase the value of *J*(**w**,H_*A*_), alternating updates to **w** and H_*A*_ guarantee convergence to a local minimum of the overall objective function (Bertsekas, 1999; Boyd and Vandenberghe, 2004). Intuition for this result can be gained by examining the sequence

$$\begin{array}{l} J\left(w^{\left(0\right)},H_{A}^{\left(0\right)}\right)\geq J\left(w^{\left(1\right)},H_{A}^{\left(0\right)}\right)\geq J\left(w^{\left(1\right)},H_{A}^{\left(1\right)}\right)\geq \cdots \\ \geq J\left(w^{\left(j+1\right)},H_{A}^{\left(j\right)}\right)\geq J\left(w^{\left(j+1\right)},H_{A}^{\left(j+1\right)}\right)\geq \cdots \end{array}$$

The solutions to both (P1) and (P2) are found numerically by searching for stationary points of the respective objective functions (see Section 4), i.e., when ∇_**w**_*J*(**w**,H_*A*_) = 0 and $\nabla_{h_{k}^{A}\left(t,f\right)}J\left(w,H_{A}\right)=0$. For the regression coefficients, upon convergence of (P1), and assuming the minimum lies within the feasible set formed by the constraints on the *w*_*k*_, the regression coefficient vector can be written as

(5) $$\begin{array}{l} w=C\langle y_{t}\cdot \left[1-\sigma \left(y_{t}w^{T}r_{t}\right)\right]\cdot r_{t}\rangle_{t} \end{array}$$

We interpret the term $\left[1-\sigma \left(y_{t}w^{T}r_{t}\right)\right]$ as a “prediction error” and consequently hard-to-predict responses have more influence on choice of the optimal regression coefficients. Moreover, because the *w*_*k*_ for *k* > 0 are constrained to be nonnegative, those coefficients can be thought of as a *population gain vector* that applies more weight to task-relevant vs. task-irrelevant neurons.

Next, upon convergence of (P2), the adapted STRFs are found as

(6) $$\begin{array}{ll} h_{k}^{A}\left(t,f\right)=h_{k}^{0}\left(t,f\right)+\frac{C}{\lambda }\cdot w_{k}\cdot m_{k}\left(t,f\right)\langle y_{t^{\prime }}\cdot \left[1-\sigma \left(y_{t^{\prime }}w^{T}r_{t^{\prime }}\right)\right]\\ \;\cdot s\left(t^{\prime }-t,f\right)\rangle_{t^{\prime }} & \end{array}$$

Equation (6) contains the main theoretical result of the Feature-Based Model and shows how STRF plasticity predicted by the proposed framework is consistent with the contrast filtering hypothesis. First, attention-induced STRF plasticity directly reflects the spectro-temporal structure and features of the (time-reversed) target and reference stimuli, as given in the averaging term. The impact of the stimulus on adaptation at each time is proportional to the difficulty of predicting its corresponding label. Second, because we have constrained the regression coefficients *w*_*k*_ for *k* > 0 to be non-negative, the behavioral meaning of the labels is preserved so that acoustic features of the target (*y*_*t*_ = +1) are guaranteed to be *enhanced* whereas those of the reference (*y*_*t*_ = −1) are *suppressed*. Third, STRF plasticity is guaranteed to be local as a consequence of multiplying the sum with the Gaussian-shaped spectro-temporal mask *m*_*k*_(*t, f*). Finally, the first term encourages stability in the STRFs by resisting change from their original shapes, the magnitude of the effect being controlled by *C* and λ.

### 2.4. Feature-based model: validation

We validate the model by simulating task-driven plasticity on a number of spectral behavioral tasks that have been explored in studies of auditory cortex. We first consider a *tone detection* task, where an animal is trained to detect an isolated tone in the context of a broadband noise reference (Fritz et al., 2003). This noise reference is referred to as a temporally orthogonal ripple combination (TORC), and is typically used in neurophysiological recordings to estimate a neuron's STRF. The second is a *chord detection* task, where an animal is trained to detect a multi-tone complex in the context of a broadband noise reference (Fritz et al., 2007c). Finally, we consider a *tone discrimination* task, where an animal is trained to detect a target tone in the context of a specified reference tone (Fritz et al., 2005c). The details of the stimuli used for each task are provided in Table 1 and the details of stimulus construction are provided in the Section 4.

**Table 1:** **Details of the tasks considered for the feature-based model**.

**Task**	**Target**	**Reference**
Tone detection	0.25, 0.5, 1, 2, 3.25 kHz	TORCs
Chord detection	0.25/0.5/0.75, 0.5/0.75/2, 0.5/1/2, 1/2/1.5, 1.75/2/3.25 kHz	TORCs
Tone discrimination	0.25, 0.25, 0.5, 0.5, 1 kHz	0.5, 1, 1, 2, 2 kHz

**Figure 3:**
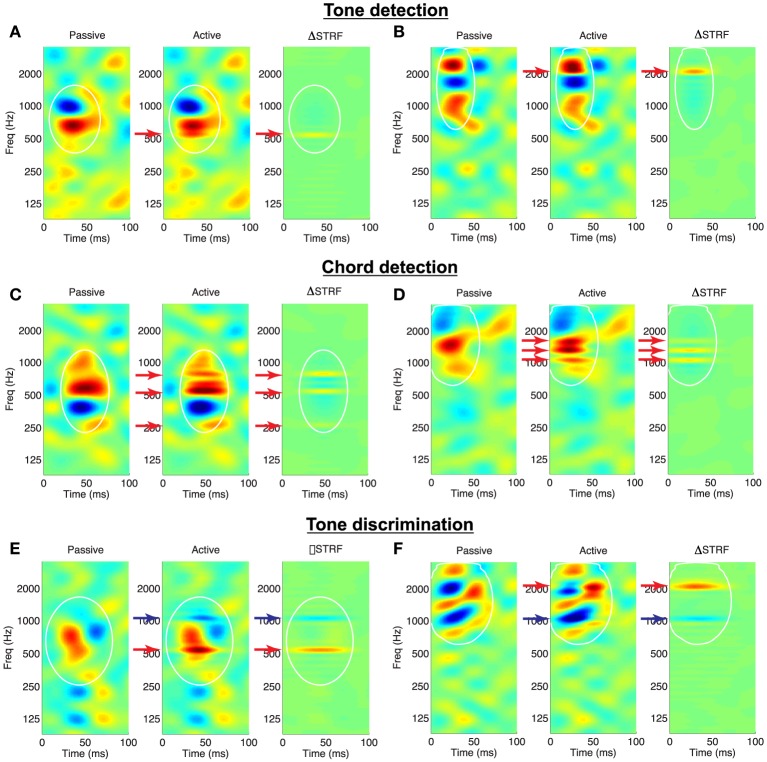
**Validation of the Feature-Based Model on a variety of behavioral tasks**. Each panel shows an STRF in the passive and active behavioral state, and the difference STRF illustrates the effects of the model on STRF shape. **(A,B)** *Tone detection:* Target tones (red arrows) elicit increased excitation at the target frequency. The difference pattern also reveals a small degree of inhibition at non-target frequencies within the mask. **(C,D)** *Chord detection:* Target tones elicit increased excitation at each of the frequencies in the target complex, with regions of suppression between and outside the targets. **(E,F)** *Tone discrimination:* Target tones elicit increased excitation whereas reference tones (blue arrows) are suppressed. White lines: isoline contours of the spectro-temporal mask at the 20% level. STRFs are interpolated for display. Examples shown for λ = 10^−4.5^, *C* = 10^−3^.

To visualize the effects of attention on the shapes of the receptive fields, we consider the difference between the Euclidean-normalized active and passive STRFs (Δ*STRF*); examples of the induced adaptation patterns for the spectral tasks are shown in Figure 3. For tone detection, Figures 3A,B illustrate that target tones (red arrows) induce local, excitatory changes in the STRFs at the target frequencies. This is apparent from the active STRFs (middle subpanels) as well as from the difference STRF (right subpanels). The difference STRF also reveals that the effect of the noise reference is to introduce a small degree of suppression within the mask and surrounding the tone. Similar effects are observed for the chord detection in Figures 3C,D: target tones induce local, excitatory changes, with suppression around and in between the target tones. Finally, shown in Figures 3E,F are example adaptation patterns for the tone discrimination task. We observe that target tones induce excitatory changes whereas reference tones (blue arrows) induce inhibitory changes in the active STRFs.

**Figure 4:**
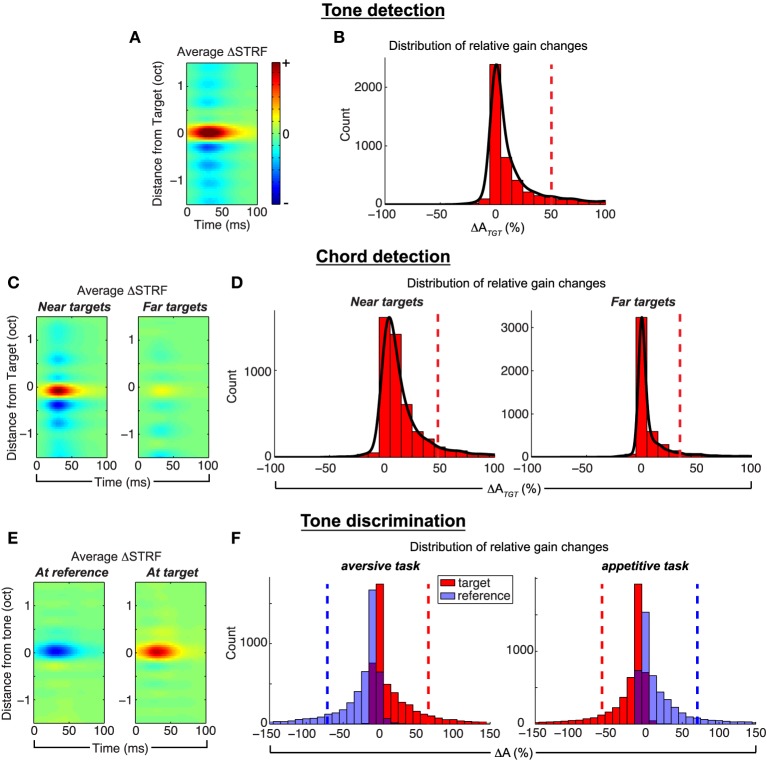
**Population analysis of the Feature-Based Model**. *Tone detection:* the average Δ*STRF* in **(A)**, computed by aligning all difference STRFs at the target frequency, shows that target tones elicit increased excitation, whereas the broadband noise reference induces suppression in areas spectrally adjacent to the target. **(B)** Shows that relative gain changes at the target due to attention are overwhelmingly excitatory. *Chord detection:* **(C)** shows the average Δ*STRF* aligned to targets nearest (left) and farthest (right) from a neuron's BF. Targets close to BF induce much larger excitatory changes than those farthest away, and this pattern is also observed in Δ*A*_*TGT*_ in **(D)**. Suppressive effects are similar to those observed in single tone detection tasks. *Tone discrimination:* **(E)** shows the average Δ*STRF* aligned at the reference and target tones in an aversive task setup. STRF changes are suppressive at the reference and excitatory at the target, which is also observed in patterns of Δ*A* (**F**, left). However, when the behavioral meaning of the target and reference is reversed, as in an appetitive task, STRF plasticity patterns are similarly reversed (**F**, right). Average Δ*STRF* patterns are interpolated for display. Dashed vertical lines denote population means. Results shown for λ = 10^−4.5^, *C* = 10^−3^.

We quantify population effects using approaches described in previous physiological studies (see e.g., Fritz et al., 2003), and the results are summarized in Figure 4. First, to visualize population effects across a number of targets (references), we compute Δ*STRF* aligned at the target (reference) frequencies, and average across all ensembles and target (reference) tones. Next, in order to quantify the size of the attentional effect, we compute the relative change of STRF gain, at the location of maximum difference in the target (reference) channel, between the passive and active settings; we refer to this as Δ*A* and subscript accordingly for each task.

For tone detection, Figure 4A shows that across all targets and ensembles, active attention simulated by the model induces local, excitatory changes in the STRFs at the target tone, with inhibitory changes spectrally adjacent to the target. Figure 4B shows that the distribution of Δ*A*_*TGT*_ is overwhelmingly excitatory (mean = +50.87±6.7% s.e.m.) with a heavy tail to the right. For each ensemble and across all targets, excitatory changes are significant (*p* ≪ 0.001, *t*-test and Wilcoxon signed-rank test). Importantly, similar observations have been made in ferret recordings by Fritz et al. (2003).

For chord detection, the target stimuli comprise three tones, some of which may be near or far to a given neuron's best frequency (BF). Based on the Gaussian shape of the mask *m*_*k*_(*t, f*) for a given filter, we expect that tones near BF would induce stronger plasticity effects compared to those far from BF. We verify this by computing the average Δ*STRF* aligned to target tones nearest to and furthest from BF, and these results are shown in Figure 4C. As shown, tones near BF induce stronger local excitatory changes compared to tones far from BF. As suggested previously in Figure 3, the suppressed sidebands surrounding the target tones show that the active STRFs were suppressed in between each of the target tones. The inhibitory effect is also relatively stronger for tones near BF compared to those far from BF. Importantly, this analysis has parallels with that of Fritz et al. (2007c), and we again find a general correspondence with those previously reported results. Finally, in Figure 4D, we consider the distribution of Δ*A*_*TGT*_ for near vs. far targets across all ensembles. These distributions show that changes at the target tones are overwhelmingly excitatory (mean +48.4±9.8% vs. +35.1±7.4%, near vs. far, s.e.m.) with heavy tails to the right, and are stronger for targets near BF vs. those far from BF. For each ensemble and across all targets, excitatory changes are significant (*p* < 0.03, *t*-test and Wilcoxon signed-rank test).

Next, for tone discrimination, we considered Δ*STRF* aligned to both the reference and target tones; these results are shown in Figure 4E averaged across all ensembles and target/reference combinations. As shown, the model induces local, inhibitory changes at the reference compared to local, excitatory changes at the target. Importantly, these differential plasticity effects are consistent with observations by Fritz et al. from a ferret study (Fritz et al., 2005c). On the left side of Figure 4F, we show the distribution of Δ*A* at the target and reference tones. As predicted by the model, attention induces excitatory changes at the target (red, mean = +66.0 ± 12.4% s.e.m.) while changes at the reference are inhibitory (blue, mean = −71.4 ± 5.0% s.e.m.). For each ensemble and across all tasks, excitatory and inhibitory changes are significant (*p* ≪ 0.001, *t*-test and Wilcoxon signed-rank test).

Finally, we verify that non-negativity constraints imposed on the regression coefficients allow the model to capture the behavioral meaning associated with the target and reference stimuli. As demonstrated by David et al. (2012) in a ferret study, differences in animal training for aversive tasks (target associated with negative reward) vs. appetitive tasks (target associated with positive reward) yield excitatory and inhibitory patterns at the target and reference tones that are flipped versions of each other. In our framework, this is achieved by simply flipping the sign of the labels associated with the target and reference stimuli. The recomputed Δ*A* distributions after flipping labels are shown on the right side of Figure 4F. As shown, the distributions of Δ*A* for the appetitive task are flipped versions of the aversive task (target mean = −59.1 ± 8.5%, reference mean = +70.5 ± 7.9%, s.e.m.). For each ensemble and across all tasks, excitatory and inhibitory changes are significant (*p* ≪ 0.001, *t*-test and Wilcoxon signed-rank test). These results confirm that the model does indeed capture aspects of task structure.

### 2.5. Object-based model: theoretical results

The Feature-Based Model, while sufficient to account for adaptation patterns in purely spectral tasks, is restricted to act at the level of the raw spectro-temporal features that characterize the task-related stimuli (see Equation 6). However, accumulating evidence suggests that top-down attention can instead modulate neural representations at the level of auditory objects (Alain and Arnott, 2000; Griffiths and Warren, 2004; Krumbholz et al., 2007; Shinn-Cunningham, 2008; Bizley and Cohen, 2013). Broadly speaking, object-based attention refers to the selective allocation of cognitive resources to an *abstracted* representation of a stimulus. For our purposes, we interpret this as attention directed toward collections of features that may be used to distinguish broad stimulus classes from one another (e.g., speech vs. non-speech sounds). One way to abstract acoustic information in a spectrogram is to consider its representation in the Fourier domain, where the strength of observed spectro-temporal modulations (i.e., the Fourier magnitude profile) could be considered separately from the relative activation of the modulations to one another (i.e., the Fourier phase profile). Thus, attention directed toward a collection of spectro-temporal dynamics, rather than the relative timings of the observed acoustics, represents an instantiation of object-driven attention. For example, in complex acoustic scenes, a listener may wish to attend to conspecific vocalizations in noisy natural environments, retuning cognitive resources to enhance responses to time-varying harmonicity cues (which are often characteristic of animal communication sounds) while suppressing those to the din of spectro-temporally broad background interference.

Furthermore, there is neurophysiological evidence suggesting that receptive field plasticity that reflects differences in stimulus modulation profiles contributes to improved performance of behavioral tasks. For example, Beitel et al. (2003) showed that the temporal modulation profiles of A1 neurons in monkeys trained to discriminate temporally modulated tone sequences adapted to enhance responses of faster target modulations (associated with a negative reward) while suppressing responses to slower reference modulations. In a visual study, David et al. (2008) found that the modulation profiles of spatio-temporal receptive fields in higher visual cortex adapted to match those of a target stimulus in both discrimination and search tasks. Finally, Yin et al. (2014) recently demonstrated that the joint spectro-temporal modulation profiles of STRFs in ferret A1 adapted to reflect the difference in modulation characteristics of upward vs. downward moving tone pips. Motivated by these examples, we sought to extend the proposed framework to circumstances where task-relevant stimuli could be discriminated based on differences in their spectro-temporal dynamics, and we directly modified STRF shapes in the Fourier domain accordingly.

We begin by first modifying the firing rate model as

(7) $$\begin{array}{l} r_{k}\left(t,f;m\right)=h_{k}^{A}\left(t,f\right){*}_{tf}s_{m}\left(t,f\right) \end{array}$$

with corresponding *modulation domain* representation

(8) $$\begin{array}{l} \left|R_{k}\left(\omega ,\Omega ;m\right)|=|H_{k}^{A}\left(\omega ,\Omega \right)|\cdot |S_{m}\left(\omega ,\Omega \right)\right| \end{array}$$

where ^*^_tf_ denotes convolution in time and frequency, and *R*_*k*_(ω, Ω; *m*), $H_{k}^{A}\left(\omega ,\Omega \right)$, and *S*_*m*_(ω, Ω) are the 2D Discrete Fourier Transforms of firing rate, STRF, and the *m*'th stimulus token, respectively. In the modulation domain, ω characterizes modulations along the temporal axis (*rate*, in Hz) whereas Ω characterizes modulations along the spectral axis (*scale*, in cycles/octave). For technical reasons (see Supplementary Text 1), in this instantiation of the model we forego use of the mask, but we address its absence later in the Section 3.

The development of the Object-Based Model development mirrors that of the Feature-Based Model. First, we form a firing rate vector as $R_{m}=\left[1,\underset{\omega \Omega }{\sum }|R_{1}\left(\omega ,\Omega \right)|,\cdots ,\underset{\omega \Omega }{\sum }|R_{K}\left(\omega ,\Omega \right)|\right]\in {\mathbb{R}}^{K+1}$. Next, we again use logistic regression and Euclidean norm to quantify the balance between discriminability and stability, and define the objective function

(9) $$\begin{array}{l} J\left(w,{\hat{H}}_{A}\right):\underset{\text{Discriminability}}{\underset{︸}{\frac{1}{2}||w|{|}_{2}^{2}-C\cdot {\langle \log\sigma \left(y_{m}w^{T}R_{m}\right)\rangle }_{m}}}+\underset{\text{Stability}}{\underset{︸}{\frac{\lambda }{2}\underset{k}{\sum }||\Delta_{k}|{|}_{F}^{2}}} \end{array}$$

where **w** is defined as before, ${\hat{H}}_{A}:={\left\{\left|H_{k}^{A}\left(\omega ,\Omega \right)\right|\right\}}_{k=1}^{K}$, and $\Delta_{k}:=|H_{k}^{0}\left(\omega ,\Omega \right)|-|H_{k}^{A}\left(\omega ,\Omega \right)|$.

To optimize Equation (9), we again used block-coordinate descent, alternating between solving two convex subproblems:

(P3) $$\begin{array}{l} \arg\underset{w}{\min}J\left(w,{\hat{H}}_{A}\right)\text{ subject to }w_{k}\geq 0,\;k=1,2,\cdots \;,K \end{array}$$

(P4) $$\begin{array}{l} \arg\underset{{\hat{H}}_{A}}{\min}J\left(w,{\hat{H}}_{A}\right)\text{ subject to }|H_{k}^{A}\left(\omega ,\Omega \right)|\geq 0,\;\forall k,\omega ,\Omega \end{array}$$

The constraints on (P4) are required since modulation profiles $\left|H_{k}^{A}\left(\omega ,\Omega \right)\right|$ are necessarily nonnegative.

Optimizing (P3) yields regression coefficients similar to those in Equation (5). Next, upon convergence of (P4), and assuming the minimum lies within the feasible set formed by the constraints on $\left|H_{k}^{A}\left(\omega ,\Omega \right)\right|$, the adapted STRF modulation profiles can be written as

(10) $$\begin{array}{l} |H_{k}^{A}(\omega ,\Omega )|=|H_{k}^{0}(\omega ,\Omega )|+\frac{C}{\lambda }\cdot w_{k}\cdot \langle y_{m}[1-\sigma (y_{m}w^{T}R_{m})]\\ \;{\cdot |S_{m}(\omega ,\Omega )|\rangle }_{m} \end{array}$$

Equation (10) contains the main theoretical result of the Object-Based Model, which is again consistent with the contrast filtering hypothesis and similar in spirit to the Feature-Based Model. First, attention-induced STRF plasticity directly reflects the spectro-temporal modulation profiles of the target and reference stimuli, as given in the averaging term. The impact of each stimulus sample on adaptation is proportional to the difficulty of predicting its corresponding label. Again, because we have constrained the regression coefficients *w*_*k*_ to be non-negative, the behavioral meaning of the labels is preserved so that acoustic features of the target are *enhanced* whereas acoustic features of the reference are *suppressed*. The first term acts to resist changes from the initial STRF modulation profile, the magnitude of the effect being controlled by *C* and λ. Finally, we note that to visualize the adapted STRFs in time-frequency, we use the original, unmodified phase of the passive STRF.

### 2.6. Object-based model: predictions

**Figure 5:**
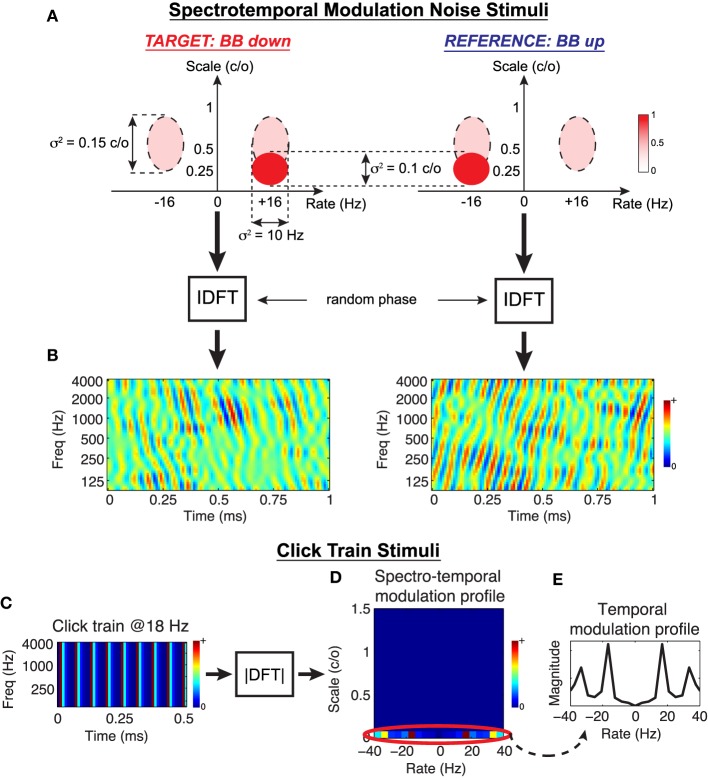
**Stimulus design for testing the Object-Based Model**. *Spectro-temporal modulation noise stimuli:* As illustrated in **(A)**, noise stimulus profiles are designed to overlap in the modulation domain over a broad range (dashed ellipses), and each class is distinguished by added energy centered at a prescribed rate and scale (solid ellipses). The modulation profiles are coupled with random phase, and an inverse 2D Discrete Fourier Transform is performed to synthesize the stimuli in time-frequency **(B)**. In this example, a target stimuli characterized by broad, downward drifting modulations is contrasted with a reference of broad, upward drifting modulations. *Click train stimuli:* Simple broadband click trains are synthesized directly in time-frequency **(C)**, and necessarily only have energy in the spectro-temporal modulation domain at 0 c/o **(D)**. For analysis purposes, we consider changes in the STRF temporal modulation profiles only at this scale **(E)**.

To evaluate the predictions of the Object-Based Model, we consider two behavioral tasks that can be readily explored in animal studies. The first is *spectro-temporal modulation noise discrimination*. Classes of natural stimuli often overlap in terms of their spectral and temporal modulation content but are distinguished by the additional presence or absence of energy at certain rates and scales, e.g., speech vs. speech+noise, conspecific vocalizations in noisy natural environments, etc. In this spirit, we synthesize complex spectro-temporal noise stimuli that share a broad range of modulations but are distinguished by additional energy at downward vs. upward rates and scales. The stimuli are generated by specifying the energy distribution of the target and reference modulation profiles, coupling them with random phase, and performing an inverse 2D Fourier transform to obtain the stimulus spectrograms. An example of this process is shown in Figures 5A,B for what we term a *Broadband Down* (BB Down) target and *Broadband Up* (BB up) reference. The ellipses in Figure 5A represent Gaussians in the modulation domain, and the dashed lines indicate the set of modulations that are shared between the target and the reference. Here the target is characterized by the addition of a range downward modulations centered at (+16 Hz, 0.25 cyc/oct) whereas the reference is a flipped version of the target, containing added upward modulations. After coupling with random phase and performing an IDFT, we obtain the spectrograms shown in Figure 5B. For this work we consider four discrimination tasks, the details of which are provided in the Section 4 and summarized in Table 2.

**Table 2:** **Details of the tasks considered for the Object-Based Models**.

**Task**		**Target**	**Reference**
Spectro-temporal modulation noise discrimination	*Narrowband Up* (NB up)	(−10 Hz, 1 cyc/oct)	(+10 Hz, 1 cyc/oct)
	*Narrowband Down* (NB down)	(+10 Hz, 1 cyc/oct)	(−10 Hz, 1 cyc/oct)
	*Broadband Up* (BB up)	(−16 Hz, 0.25 cyc/oct)	(+16 Hz, 0.25 cyc/oct)
	*Broadband Down* (BB down)	(+16 Hz, 0.25 cyc/oct)	(−16 Hz, 0.25 cyc/oct)
Click rate discrimination		18 Hz	5 Hz
		24 Hz	7 Hz
		32 Hz	9 Hz

The second task we consider is *click rate discrimination*, where the goal is to discriminate a fast from a slow click train. To the best of our knowledge, there have been no studies that report population STRF plasticity patterns for this task (though see the examples presented in Fritz et al., 2005c). We synthesize idealized click trains directly in the time-frequency domain, and an example is shown in Figure 5C with its corresponding spectro-temporal modulation profile shown in Figure 5D. By construction, the broadband clicks contain energy only at 0 cyc/oct, i.e., purely temporal modulations. Consequently, adaptation of the modulation profiles will only occur at this scale (Figure 5D, circled), so we restrict our population analysis accordingly. Figure 5E shows the temporal modulation profile of an example stimulus, with a peak at 18 Hz and associated harmonics. For this work, we consider three click discrimination tasks, and the specific details of the stimuli are provided in the Section 4 and summarized in Table 2.

**Figure 6:**
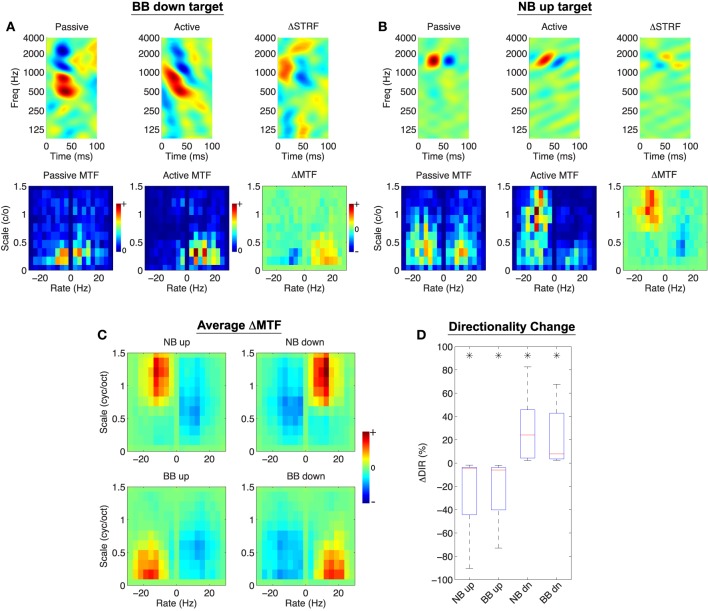
**Object-Based Model predictions for spectro-temporal modulation noise discrimination**. In **(A,B)**, the top row shows the passive, active, and difference STRF, whereas the bottom row shows the passive, active, and difference MTF (note: Δ*MTF* is *not* the modulation profile of Δ*STRF*). The active STRFs are characterized by downward or upward changes in orientation depending on the target stimulus class (**A,B**, respectively). Furthermore, the difference MTF illustrates that target modulations are enhanced whereas reference modulations are attenuated. **(C)** Shows that across all tasks and populations, target modulations are enhanced and reference modulations are suppressed. Finally, **(D)** shows that changes in directional preference of the active STRFs, as quantified by Δ*DIR*, reflect a significantly increased sensitivity to the target class (^*^:*p* < 0.01), Wilcoxon signed-rank test. Results shown for λ = 10^−4^, *C* = 0.5.

For spectro-temporal modulation noise discrimination, we find model-induced adaptation patterns for individual neurons that are consistent with the contrast filtering hypothesis. Shown in Figures 6A,B are two examples of the model effects when engaged in two modulation noise discrimination tasks. As before, the top rows of each panel show the passive, active, and normalized difference STRF. Here, however, the bottom rows show the passive, active, and difference modulation transfer functions (Δ*MTF*) over a broad range of rates and scales. In both examples, the model predicts STRF plasticity that reorients and sharpens tuning for target modulations (downward for Figure 6A, upward for Figure 6B). This effect is also clear from the difference MTFs, which show explicit enhancement of target and suppression of reference modulations.

We also find that population patterns of plasticity are broadly consistent with the contrast filtering hypothesis. We summarized these population patterns in the modulation domain by averaging Δ*MTF* across all neurons; these results are shown in Figure 6C. For each task, we find that on average target modulations are enhanced whereas reference modulations are suppressed. We also consider model effects on the directional preference of the STRFs, as quantified by a directionality index (*DIR*, see Section 4). In general, positive *DIR* indicates a preference for downward modulations whereas negative *DIR* indicates a preference for upward moving modulations. The effect of the model between the passive and active settings can be measured by computing the change in directionality, defined as Δ*DIR*: = *DIR*_*A*_−*DIR*_*P*_ (where the subscripts denote active and passive, respectively). Thus, positive values of Δ*DIR* indicate a shift toward a preference for downward modulations, whereas negative values of Δ*DIR* indicate a shift toward a preference for upward moving modulations. Figure 6D shows the distributions of Δ*DIR* for each tasks. As shown, upward moving targets induce a significant directional preference for upward modulations, and similarly so for downward moving targets (*p* < 0.01, Wilcoxon signed-rank test).

**Figure 7:**
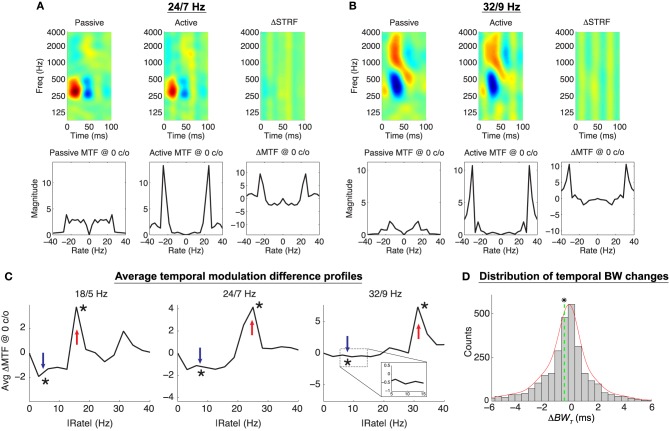
**Object-Based Model predictions for click rate discrimination**. In **(A,B)**, the top row shows the passive, active, and difference STRF, whereas the bottom row shows the passive, active, and difference MTF (note: Δ*MTF* is *not* the temporal modulation profile of Δ*STRF*). The active STRFs are characterized by the addition of broadband temporal ripples that have the effect of slightly narrowing or broadening the main excitatory subfields of the STRFs (**A,B**, respectively). The difference MTFs at 0 c/o show enhancement and suppression of the target and reference click rates, respectively. **(C)** shows the average Δ*MTF* at 0 c/o, folded at 0 Hz for clarity, for each of the click rate discrimination tasks, showing significant enhancement and suppression at the target and reference click rates, respectively (^*^p ≈ 0, Wilcoxon signed-rank test). **(D)** shows the distribution of changes in temporal bandwidth for STRF main excitatory subfields across all tasks and ensembles. Temporal bandwidth, while on average slightly decreased [mean Δ*BW*_*T*_ = −0.53 ms, dashed vertical line; (^*^:*p* < 0.01), *t*-test], can be both increased and decreased by adaptation of the temporal modulation profile. Results shown for λ = 10^−4^, *C* = 0.5.

For click rate discrimination, we find that the Object-Based Model induces plasticity patterns in individual neurons that are consistent with the contrast filtering hypothesis, with effects that are evident in both the original time-frequency space as well as in the temporal modulation profiles. As expected, we find that modulations at the target click rate are enhanced, whereas modulations at the reference click rate are suppressed. Shown in Figures 7A,B are two examples of the simulated plasticity effects for this task. The top row of each panel shows the passive, active, and normalized difference STRF (Δ*STRF*) whereas the bottom row shows the passive, active and difference modulation transfer functions (Δ*MTF*) at 0 cyc/oct. For both examples, it is clear in the time-frequency domain that the model induces purely temporal adaptation, as evidenced by the vertical bars in Δ*STRF*. These changes had an apparent effect on the temporal bandwidth of the main excitatory subfield of the active STRFs, in some cases inducing a narrowing and in others a broadening of the subfields (Figures 7A,B, respectively). Furthermore, in the modulation domain, it is clear from the difference MTFs that energy at the target rate is enhanced whereas energy at the reference click rate is suppressed.

We again find that population patterns of plasticity are broadly consistent with the contrast filtering hypothesis, with adapted neurons exhibiting increased (decreased) sensitivity to the target (reference) click rates. To summarize these population patterns in the modulation domain, for each task we first averaged Δ*MTF* across all neurons and for clarity fold the modulation profile about 0 Hz rate; these results are shown in Figure 7C. As shown, for each task Δ*MTF* is positive at the target click rate (and its harmonics) and negative at the reference click rate. Changes at the target and reference click rates are significant for each task (*p* ≈ 0, Wilcoxon signed rank test). In Figure 7D we also show the distribution of changes in the temporal bandwidth of the main excitatory subfields (Δ*BW*_*T*_, see Section 4). Here, negative values indicate temporal narrowing whereas positive values indicating temporal broadening. Temporal bandwidth in the active STRFs tends to be slightly, but significantly, narrowed (mean Δ*BW*_*T*_ = −0.53 ms, *p* < 0.01, *t*-test). However, the distribution shows that while the changes are generally are quite subtle, a large number of neurons (40.4% across all tasks and ensembles) have an absolute change greater than 1 ms. Interestingly, the model predicts that excitatory subfields will both contract and expand as needed to enhance sensitivity to target click modulations, as indicated by both negative and positive values of Δ*BW*_*T*_. Similar behaviors have been observed in neurophysiological studies yet to be published (Fritz et al., 2005b), though an exact quantification of this effect in experimental findings is not yet readily available.

## 3. Discussion

In this study, we proposed and explored a discriminative framework for modeling task-driven plasticity in auditory cortical receptive fields. The framework predicts STRF adaptation patterns that are consistent with the contrast filtering hypothesis: that neural tuning characteristics at the level of primary auditory cortex adapt to enhance acoustic features of the foreground while actively suppressing those of the background. An important contribution of this framework is a set of predictions for temporal and spectro-temporal tasks for which experimental data is not readily available or confirmed. Furthermore, as we explore below, the model has a modular structure that has a number of parallels with neural circuits speculated to be engaged during attentional tasks.

We proposed two instantiations of the framework: a Feature-Based Model that acts directly based on raw acoustic features in the time-frequency domain; and an Object-Based Model that acts in a stimulus phase-invariant fashion on an abstracted representation of the stimuli in the spectro-temporal modulation domain. We showed, via simulations of a number of spectral behavioral tasks, that the Feature-Based Model induced localized STRF adaptation that enhanced representation for the target tone while inducing mild sideband suppression (for tone/chord detection tasks) or narrowband suppression at the reference tone (for tone discrimination tasks). Importantly, these results are consistent with plasticity patterns previously reported in neurophysiological studies (Fritz et al., 2003, 2005c, 2007c). We also showed, via the tone discrimination tasks, that switching the behavioral meaning associated with target and reference stimuli (i.e., by switching the model labels) induces opposite plasticity patterns. This is akin to modifying animal training protocol from an aversive to appetitive task structure where similar flipped adaptation patterns have been observed in ferret A1 neurons (David et al., 2012). This suggests that the model captures aspects of task structure, which has yet to be explicitly accounted for by previous computational models (Mesgarani et al., 2010; David et al., 2012).

Next, we explored predictions of the Object-Based Model on tasks that could be readily evaluated in neurophysiological studies. We first considered the task of spectro-temporal modulation noise discrimination. This was intended to model naturalistic scenarios where a listener seeks to direct attention among acoustic classes of similar timbres, i.e., those that share a broad range of spectro-temporal modulations but differ based on the presence or absence of energy at a smaller set of rates and scales. For these stimuli, the model predicted enhancement at the subset of modulations that defined the target class, whereas we observed suppression at the subset of modulations that defined the reference class. The overall effects in time-frequency were an effective reorientation and sharpening of the STRFs to the target modulations, and we quantified these changes using a directionality measure that characterized a neuron's preference for downward vs. upward drifting modulations.

Finally, we considered the task of click rate discrimination for which, to the best of our knowledge, population patterns of STRF plasticity have yet to be reported (save for examples reported by Fritz et al., 2005a). The model predicted that for purely temporal tasks, the temporal modulation profile of the active STRFs is enhanced at the target click rate and suppressed at the reference click rate. This had the effect of introducing broadband, temporal ripples in time-frequency, as evidenced by the difference STRFs in Figures 7A,B. While it has previously been observed in other animal models and temporal tasks that the temporal dynamics of cortical neurons can shift to become more responsive (i.e., reduced temporal bandwidth or latency) (Kilgard and Merzenich, 1998; Kilgard et al., 2001; Fritz et al., 2005a), the Object-Based Model predicts that the main excitatory subfields of neurons can become either temporally narrower or broader so long as the overall temporal modulation profile is suitably adapted at the target and reference click rates.

It is also worth noting that our model predictions, especially for the temporal tasks, are consistent with observations from studies beyond the ferret animal models focused on in this work. In particular, the results of Bietel et al. and Bao et al. both highlight that task performance can influence temporal firing patterns in a manner that reflects the temporal statistics of a target stimulus associated with a positive reward (Beitel et al., 2003; Bao et al., 2004).

### 3.1. An integrated framework for modeling attention-driven plasticity

Optimization within the proposed framework is by necessity constrained and iterative, due the need to alternate between solving two convex subproblems to determine optimal regression coefficients and STRF parameters. However, this approach may reflect an analogous iterative adaptation strategy among neural circuits in the cortical hierarchy thought to be involved in task-driven auditory attention. In particular, it has been suggested that attention involves an iterative circuit among basal forebrain, prefrontal cortex (PFC), and sensory cortex that, from a computational perspective, has a number of parallels with our proposed framework (Fritz et al., 2005a; Rasmusson et al., 2007; Shamma et al., 2010).

**Figure 8:**
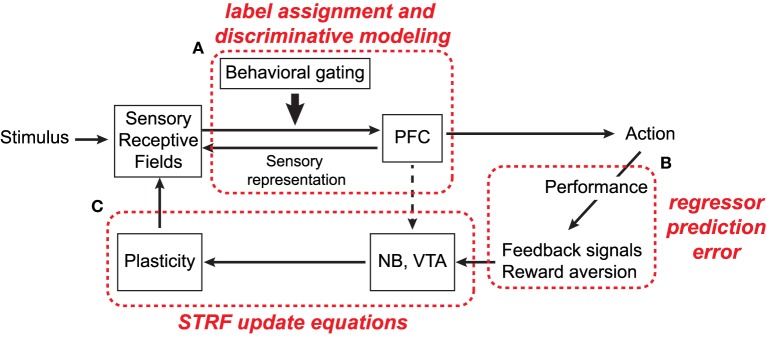
**Simplified schematic of anatomical circuits thought to be involved in attention-driven auditory cortical plasticity (adapted from Shamma et al., 2010)**. Refer to text for details.

A simplified schematic of this process is shown in Figure 8 and the basic process is as follows. Input acoustic stimuli are processed by a bank of cortical receptive fields, which project to, and receive projections from, executive control networks in PFC. Importantly, projections to PFC are gated according to behavioral and task salience, i.e., only task-relevant signals are passed along and processed (Fritz et al., 2010). Decoded signals in PFC in turn cause motor responses which prompt the listener to act (e.g., cease licking water in response to a target tone), and consequently induce plasticity to improve performance of the task. These feedback circuits likely involve nucleus basalis (NB) and the ventral tegmental area (VTA), basal forebrain areas which have been implicated in cortical plasticity (Bao et al., 2001; Kilgard et al., 2001).

As annotated in the figure, we propose that the framework described in this study has useful parallels with the circuits enclosed within the dashed boxes and that, in general, the alternating optimization procedure reflects a biologically plausible strategy for fine-tuning sensory input based on task-performance. In particular, we argue that during active attention, the computational goal of top-down executive control circuits, like those in PFC, is to assign behaviorally meaningful categorial labels to observed ensemble responses in primary auditory cortex (box A). Subsequent classification decisions in turn induce appropriate motor responses to perform the task at hand. Furthermore, as seen in Equations (6) and (10), the magnitude of plasticity effects in the model is directly proportional to the magnitude of regressor prediction errors (box B). This has parallels with behavioral results in ferret studies, where the magnitude of STRF plasticity effects is directly correlated with an animal's ability to successfully perform a task (Fritz et al., 2003; Atiani et al., 2009). Finally, the extent to which acoustic features of the foreground and background are enhanced and suppressed, respectively, is governed by the STRF parameter update equations (box C). This may have parallels with neurotransmitters from NB and/or VTA and how they shape STRF sensitivity to specific target frequencies or spectro-temporal modulations.

As we have explored in this study, STRFs have been instrumental in understanding changes in the processing of neurons when a listener is engaged in an auditory attentional task. In particular, the observed receptive field plasticity patterns have shed light on which acoustic features are enhanced or suppressed as a function of task. Inference about corresponding biological changes at the synaptic level is limited, however, since STRFs are functional linear models of auditory signal processing whose parameters do not map directly to those of detailed synaptic models. Nevertheless, to better understand how these functional changes relate to spiking patterns in a biological neural network, it is common to consider STRFs as part of a cascade of linear, nonlinear, and feedback processes, referred to broadly as a generalized linear model (GLM) (Paninski, 2004; Truccolo et al., 2005; Calabrese et al., 2011). While GLMs do not map directly to detailed neurophysiological models, they involve components that better capture the nuances of biological spiking neurons including feature detection via a linear receptive field, nonlinear relationships between stimulus and firing rate, spike generation via Poisson point processes, and post-spike refractory or burstiness periods (see e.g., Brette and Gerstner, 2005; Ostojic and Brunel, 2011). It is our belief that the discriminative model of attention proposed in this paper can be incorporated with such GLMs as a way to better understand the impact of attention on biological spiking neural networks.

### 3.2. Relationship between the feature- and object-based models

Except for use of a spectro-temporal mask and same choice of neural firing rate model (i.e., 1D vs. 2D convolution), the receptive field adaptation mechanisms predicted by the Feature- and Object-Based Models are at their core comparable. This is clear by directly comparing the STRF update equations given in Equations (6) and (10), where the primary difference between the two is the use of stimulus phase during adaptation. More generally, to distinguish object- from feature-based attention, we considered separately the spectro-temporal magnitude and phase profiles of the observed acoustics. This allowed us to quantify the notion that object-based attention requires that cognitive resources be directed toward an *abstracted* representation of task-relevant sound classes, represented here by the collection of modulations that comprise the acoustic foreground. However, object-based attention is certainly not restricted to act merely on the Fourier domain representation of sound, since attention can act at even higher levels of abstraction, for example, by being directed to an individual melody in an orchestra, the prosodics of a target speaker at a cocktail party, or a bird watcher listening for a specific species call in nature. Furthermore, while we have drawn a clear distinction between the notions of feature- and object-based attention, the existence of such a clear difference between the two is still the subject of debate (see e.g., Krumbholz et al., 2007 and Shinn-Cunningham, 2008). Nevertheless, the proposed framework provides a means to evaluate both hypotheses as more physiological and behavioral results become available.

For the Object-Based Model, the choice of 2D convolution for modeling neural firing rate was motivated by prior work that suggests that such a representation is sufficient to capture a variety of aspects of sound perception such as speech intelligibility and timbre representation (Elhilali et al., 2003; Chi et al., 2005; Patil et al., 2012). Of course, it may be possible to adapt the STRF modulation profiles using a 1D firing rate model. However, we feel that our 2D formulation is simpler, intuitive, and, more importantly, reflects the fundamental mechanism implied by neurophysiological studies, namely, that STRFs reorient themselves to act similar to a contrast matched filter in the Fourier domain for complex spectro-temporal tasks (David et al., 2008; Yin et al., 2014).

Under what circumstances does a listener employ the Feature- and Object-based models? We hypothesize that this decision depends on task, and that the final choice is made empirically based on the behavioral outcomes of either strategy. Again, the key distinction between the two models is the use of stimulus phase in the STRF update Equations (6) and (10). So for tasks where exploiting differences in phase is important, like tone discrimination (spectral phase) or speech recognition (temporal phase), the Feature-Based Model will be employed. Conversely, for tasks where the task-relevant classes are distinguished largely based on differences in spectro-temporal modulation profiles, as with conspecific vocalizations vs. ambient environmental noise, the Object-Based Model will be employed. Of course, it is also possible that the predictions of both models, coupled with other sources of contextual information, are combined to make an overall decision. Future work should explore how exactly to combine the models, as well as how to quantify and incorporate context into the current framework.

### 3.3. Related work

Our framework was conceived in the spirit of the approaches of Mesgarani et al. (2010) and David et al. (2012), where they proposed discriminative cost functions that quantified the computational goal of task-related plasticity in the auditory system. However, as discussed earlier, these models lacked two important components: (1) a guarantee that optimal solutions capture task-valence (i.e., when the behavioral meaning of target and reference are flipped, the direction of plasticity is also flipped) and (2) the ability to adapt STRFs based on an abstracted representation of the stimulus. Our framework directly addresses these issues, predicting a qualitative correspondence with existing physiological data, and addressing stimulus phase-invariant adaptation of STRFs via their modulation profiles—which, interestingly, has also been observed in visual cortical area V4 (David et al., 2008).

More generally, however, a strong connection exists between our framework and general strategies for top-down attention and plasticity in the visual system. In particular, a recently proposed model by Borji and Itti (2014) describes an optimal attention framework that accounts for a variety of attention-driven plasticity effects in visual cortex for discrimination and search tasks, and yields predictions that qualitatively explain a broad range of attentional mechanisms that depend on task type and difficulty. Their framework is based on deriving a set of filter gain and tuning parameters that optimize a task-dependent objective function (e.g., discrimination or visual search) and prescribing an appropriate optimization procedure. The Borji and Itti vision model shares a lot of parallels with the proposed scheme that was mainly inspired by task-driven effects observed in mammalian A1 and tailored to the particularities of the auditory system. In particular, the Object-Based model paid special attention to the notion of spectro-temporal modulations and how they might be considered separately from their relative activation in an observed spectrogram. Such a distinction is unique to the auditory system, where the constituent parts of an auditory object are not necessarily well understood and still subject to debate (Griffiths and Warren, 2004; Krumbholz et al., 2007; Shinn-Cunningham, 2008; Bizley and Cohen, 2013). Nevertheless, the strong concordance between the two frameworks on the basis of a discriminative cost function for task-driven plasticity suggests that such a principle applies broadly across different sensory modalities.

### 3.4. Other model considerations

For both models presented here, we selected the hyperparameters (*C*, λ) based on what we felt yielded a reasonable correspondence with published or expected physiological results. However, just as average plasticity patterns observed in animal studies vary based on factors like motivation, level of alertness, and satiation, the plasticity patterns predicted by the model vary with choice of (*C*, λ) (see Supplementary Figure 1). The specific values of these coefficients are not critical (since they depend on factors like the amount of stimulus used and normalization applied to the STRFs and stimuli), but their importance from a modeling perspective is that they provide a mechanism for trading off between the neurophysiologically implied coding heuristics of discriminability and stability. Specific values of these parameters could be determined using cross-validation on available behavioral results and measured passive/active STRFs, but this is beyond the scope of this study.

We have interpreted the notion of a contrast filter as referring to adaptation of primary cortical receptive fields that selectively enhance and suppress acoustic features of the foreground and background, respectively (Fritz et al., 2007c, 2013). This is captured in our model by the addition of nonnegativity constraints on the regressor coefficients. In the development of the Object-Based Model, we noted that the spectro-temporal mask—which guaranteed local plasticity in the Feature-Based Model—was omitted for technical reasons (see Supplementary Text 1). In short, including a mask in the firing rate model introduces a sign ambiguity in the gradient w.r.t. the modulation profiles and as a result, even with nonnegativity constraints on the regressor, we are no longer guaranteed that target modulations will be enhanced and reference modulations suppressed. Thus, plasticity predicted by this version of the model is not strictly consistent with our interpretation of the contrast filtering hypothesis. However, data from Atiani et al. (2014) suggest that while on average target (reference) responses are enhanced (suppressed), there are many instances at synapses from A1 through prefrontal areas where opposite patterns are observed (i.e., target responses suppressed and *vice versa* for reference responses). This may reflect similarly reversed underlying receptive field plasticity patterns. Thus, just because the model enforces constraints that guarantee strict consistency with the contrast filtering hypothesis, versions of this model without such constraints will still yield interpretable results, with a modular model structure that can be mapped to circuits likely involved in attention as described earlier in the Section 3.

### 3.5. Applications and model extensions

The tuning properties of the STRFs considered in this study were varied to improve discrimination between two acoustic classes subject to biologically plausible constraints. Because these changes enhanced representation of target sounds while actively suppressing the response to reference sounds, this makes the framework attractive for application to automated sound processing systems that handle noisy or highly confusable signals. We feel that the framework presented in this study, and its parallels with anatomical circuits likely involved in attention-driven plasticity, provides a biologically sound justification for using discriminative models to induce adaptation as part of front-end feature extraction strategies. For example, possible signal processing applications include adaptive front ends for enhanced detection of speech in noisy environments, suppression of anomalous non-target sounds, and reducing confusion between pairs of phonetic classes for automatic speech recognition.

The framework can be extended in a number of ways. First, instead of varying the shapes of the raw STRFs (i.e., each time-frequency or modulation profile bin), it may be advantageous to adapt parametric representations of STRF processing based on Gabor filters (see e.g., Ezzat et al., 2007; Bellur and Elhilali, 2015). Since the optimization considered in this study takes place over tens of thousands of parameters, adapting a simpler representation that contains far fewer parameters will enable the framework to scale to large data sets and more complex tasks.

Second, because auditory scene analysis generally involves complex sounds mixtures involving many sound classes, it is also of interest to consider plasticity of STRF ensembles for discrimination problems beyond two categories. The linear discriminative model considered here was attractive largely because of its interpretable results, but extensions to multiple classes can be achieved using multi-class logistic regression or nonlinear multi-layered perceptrons. However, it remains to be seen whether induced plasticity in these settings would be consistent with the contrast filtering hypothesis and to what extent model predictions would correspond to neurophysiological results, which are unavailable to the best of our knowledge.

Third, a further avenue of exploration would be to consider ways to incorporate knowledge of unlabeled stimuli as part of the STRF plasticity process. Intuitively, unlabeled samples that are acoustically similar to available labeled examples are likely to be from the same class and thus may be used as a proxy for labeled data. General approaches from the field of semi-supervised learning quantify this notion and can likely be adapted in the context of our model. Importantly, these methods make important assumptions about the relationship between known labeled examples and new, unlabeled observations. For example, it is common to assume that observed stimuli exist in a low-dimensional subspace such that one can exploit local geometry to cluster similar observations (e.g., Belkin and Niyogi, 2004), or that one should maximize uncertainty about the unlabeled stimuli in concert with a suitable prior when updating model parameters (Grandvalet and Bengio, 2004; Erkan and Altun, 2010).

Lastly, it is also likely that the discriminability heuristic considered here is only part of the overall strategy by the auditory system to yield noise-robust representations of sound. Representation within primary auditory areas (and beyond) seem to be inherently noise robust, so it is of interest to explore the impact of introducing a robustness term into the objective function (Mesgarani and Chang, 2012; Mesgarani et al., 2014).

## 4. Methods

### 4.1. Stimuli and auditory periphery analysis

Stimuli used in the Feature-Based Model included single tones, multi-tone complexes, and spectro-temporally rich broadband noises referred to as a temporally orthogonal ripple combinations (TORCs); these noise stimuli are commonly used to drive neurons in mammalian primary auditory cortex to derive STRFs (Klein et al., 2006). We used a computational model of mammalian auditory periphery to obtain time-frequency representations for the tone and TORC stimuli referred to as auditory spectrograms (Chi et al., 2005). This model accounts for a number of stages of peripheral processing from the cochlea through auditory midbrain. First, an input signal is processed by a bank of 128 gammatone-like filters uniformly spaced along the logarithmic tonotopic axis, starting at 90 Hz, and spread over 5.3 octaves. Next, a first-order difference along frequency is followed by half-wave rectification in order to sharpen auditory responses. Finally, the responses are smoothed in time using an exponentially decaying filter with a 10 ms time constant to model short-term integration and the loss of phase locking in the midbrain. To reduce the number of parameters in the optimization described later, the spectral axis was resampled from 128 to 50 tonotopic channels spanning 5.3 octaves. This resulted in spectrograms with a spectral sampling rate of 9.4 cycles/octave and temporal sampling rate of 100 Hz.

For the Object-Based Model, we generated idealized stimuli directly in the time-frequency domain. For the temporal tasks, simple click trains were generated by spacing vertical bars at the prescribed click rate for a given task, and the bars were smoothed in time with a decaying exponential window with a 10 ms time constant. This smoothing helped to spread out temporal modulation energy, rather than having all of the temporal modulation focused solely at the prescribed click rate and its harmonics.

For the spectro-temporal tasks in general, the stimuli were designed directly in the modulation domain, coupled with random phase, and an inverse Discrete Fourier Transform was performed to obtain the spectrograms in time-frequency; this process is illustrated in Figures 5D,E. We constructed four classes of noise stimuli, referred to as *Narrowband Up* (*NB Up*), *Narrowband Down* (*NB Dn*), *Broadband Up* (*BB Up*), and *Broadband Down* (*BB Dn*). The *BB Up* and *BB Dn* classes shared energy over range of modulations defined by Gaussians centered at (±16 Hz, 0.5 c/o), and the classes were distinguished by added energy defined by a Gaussian centered at (+16 Hz, 0.25 c/o) and (−16 Hz, 0.25 c/o), respectively. The ratio of the Gaussian peaks between target to shared modulations was 2:1. The *NB Up* and *NB Dn* classes were designed similarly, except the shared modulations were centered at (±10 Hz, 0.5 c/o). The variances of the Gaussians are as specified in Figure 5D.

### 4.2. Auditory cortical receptive fields

We considered an ensemble of 2145 STRFs estimated from recordings from non-behaving ferret primary auditory cortex in response to TORC stimuli (Klein et al., 2006). The STRFs spanned 5 octaves in frequency over 15 channels (spectral sampling rate of 3 cycles/octave), with base frequencies of 125, 250, or 500 Hz. Furthermore, the STRFs spanned 250 ms in time over 13 bins (temporal sampling rate of 52 Hz).

We modified the STRFs (1) so that we had finer spectral sampling compared to the original coarse 15 channels of coverage and (2) for convenience so that the frequency range of the STRFs aligned with the output of the auditory peripheral model. To this end we assumed the base frequency of each STRF to start at 90 Hz, and resampled the spectral axis so that the STRFs spanned 5.3 octaves over *F* = 50 channels. We used cluster analysis (described previously in Carlin and Elhilali, 2013) to verify that shifting the base frequency of each STRF was not unreasonable since examples from each cluster could be found at each original base frequency (data not shown). We also resampled the temporal axis to span 250 ms over *T* = 25 temporal bins, again to gain finer temporal sampling compared to the original STRFs. Thus, each STRF can be viewed as an image patch *h*(*t, f*) ∈ ℝ^50 × 25^, with a spectral sampling rate of 9.4 cycles/octave and a temporal sampling rate of 100 Hz.

In general, the ensemble formed a richly structured representation of natural sounds, exhibiting sensitivity to localized, spectral, temporal, and joint spectro-temporal acoustic events (Theunissen et al., 2000). We also found that the ensemble contained a large number of “noisy” STRFs, i.e., shapes that appeared unconverged or had no clear preferred spectro-temporal tuning. We used a two-step procedure to remove these noisy STRFs. First, all STRFs were sorted according to the SNR associated with each recording and an initial subset was selected keeping STRFs that had an SNR of at least 2.4 dB. Next, we sorted this subset according to a separability index *SPI* ∈ [0, 1], defined as $SPI:=1-\sigma_{1}^{2}∕\underset{j}{\sum }\sigma_{j}^{2}$, where σ_*i*_ is the *i*'th singular value for a given STRF (Depireux et al., 2001). An earlier study (Carlin and Elhilali, 2013) found that *SPI* was useful for characterizing clean vs. noisy STRFs, with clean, well-structured STRFs having small *SPI* and noisy STRFs having large *SPI*. Using this measure, we removed STRFs with *SPI* ≥ 0.5, yielding an approximately “de-noised” ensemble of 810 STRFs. Finally, from this subset, we randomly selected 10 ensembles of size *K* = 100 STRFs and considered these as the initial ensembles $H_{0}=\left\{h_{k}^{0}\left(t,f\right)\right\},k=1,2,\cdots \;,K$ for this study.

Lastly, upon ensemble construction, we modeled the notion of a neuron having a finite spectral and temporal integration window by incorporating a spectro-temporal mask in the definition of neural firing rate. For each STRF, a mask was automatically determined by a least-squares fit of a non-oriented Gaussian envelope to a thresholded (at 0.75 standard deviations) and fully rectified STRF.

### 4.3. Optimization and implementation details

All simulations and analysis in this study were performed using MATLAB. Although subproblems (P1–P4) posed for each model were convex, it was not possible to determine the optimal regression coefficients and STRFs (or STRF modulation profiles) in closed form, necessitating the use of numerical techniques. The optimal parameters for the Feature-Based Model were found using fmincon function in the MATLAB optimization toolbox. We used 5 s of audio for both the target and reference stimuli. The optimal parameters for the Object-Based Model were determined using CVX, a package for specifying and solving convex programs (Grant and Boyd, 2008, 2014). For this model, we scaled each stimulus token to have unit Euclidean norm, as this seemed to improve optimization convergence. We used 75 tokens, each 250 ms in length, for both the target and reference stimuli. We run each algorithm until the relative change in the objective function is small (threshold of 10^−6^ for the Feature-Based Model and 10^−4^ for the Object-Based Model) or a maximum number of iterations is reached (30 for the Feature-Based Model, 10 for the Object-Based Model).

### 4.4. Feature-based model analysis

In line with previous neurophysiological studies (see e.g., Fritz et al., 2003), we quantified the effect of model-induced plasticity on the receptive fields by computing the difference between Euclidean-normalized active and passive STRFs (Δ*STRF*). This allowed us to directly visualize changes in STRF shape, and Δ*STRF* was aligned to the target (or reference) tone frequencies to visualize average population patterns across different tasks. We also derived a measure of relative gain change (Δ*A*) from the difference STRF at task-related frequency channels. This was computed as the relative change in (normalized) active and passive STRF magnitudes at the location of absolute maximum in Δ*STRF* at a particular target or reference channel.

### 4.5. Object-based model analysis

For the Object-Based Model, we also considered Δ*STRF* defined above to visualize changes between the active and passive STRFs. To visualize model-induced changes in the spectro-temporal modulation profiles, we considered the difference between the modulation transfer functions of the active and passive STRFs (Δ*MTF*). Average population changes could be visualized in this domain regardless of individual STRF shape and phase (David et al., 2008; Yin et al., 2014). For the click rate discrimination task in particular, all changes in the modulation domain occurred along the rate axis at a scale of 0 cyc/oct due construction of the click train stimuli. For this reason, we considered changes in modulation profile only at this scale in our analysis.

In addition to change in the modulation domain, we sought to characterize STRF changes observed in the time-frequency domain. For spectro-temporal modulation noise discrimination, the model induced clear changes in STRF orientation and directional tuning, so we employed a directionality measure (*DIR*) to characterize the degree to which a neuron was sensitive to downward vs. upward drifting modulations (Depireux et al., 2001). Directionality was defined as *DIR* = (*E*_1_−*E*_2_)∕(*E*_1_+*E*_2_), where *E*_1_ is the energy in the right-hand plane of the modulation profile, i.e., $E_{1}=\underset{\omega ,\Omega >0}{\sum }|H\left(\omega ,\Omega \right)|$ and similarly so for *E*_2_ but for negative rates. *DIR* ranges between [−1, +1], with large positive values indicating sensitivity to downward modulations, and large negative values indicating sensitivity to upward modulations. Finally, to quantify model-induced change in directional tuning, we report the difference in directionality between active and passive settings, defined as Δ*DIR* = *DIR*_*A*_−*DIR*_*P*_. Positive changes in Δ*DIR* indicate a shift toward sensitivity to downward modulations, and negative changes indicate a shift toward sensitivity to upward modulations.

For click rate discrimination, the model appeared to induce subtle changes in the temporal bandwidth of the STRF main excitatory subfield in the time-frequency domain. We extracted this temporal bandwidth in a simple non-parametric fashion as follows. First, the STRF was interpolated (by zero-padding in the modulation domain) and thresholded at two standard deviations to keep significant peaks. Next, the STRF was half-wave rectified and bounding boxes determined for islands of excitatory activity that exceeded threshold. The main excitatory subfield was defined as that which contained the neuron's best frequency/best latency peak, and temporal bandwidth was defined as the temporal width of the corresponding bounding box.

### Conflict of interest statement

The authors declare that the research was conducted in the absence of any commercial or financial relationships that could be construed as a potential conflict of interest.

## References

[B1] Aertsen A. M. H. J. Johannesma P. I. M. (1981). The spectro-temporal receptive field. Biol. Cybern. 42, 133–143. 10.1007/BF00336731 7326288

[B2] Ahveninen J. Hämäläinen M. Jääskeläinen I. P. Ahlfors S. P. Huang S. Lin F. H.. (2011). Attention-driven auditory cortex short-term plasticity helps segregate relevant sounds from noise. Proc. Natl. Acad. Sci. U.S.A. 108, 4182–4187. 10.1073/pnas.1016134108 21368107 PMC3053977

[B3] Alain C. Arnott S. R. (2000). Selectively attending to auditory objects. Front. Biosci. 5, D202–D212. 10.2741/alain 10702369

[B4] Atiani S. David S. V. Elgueda D. Locastro M. Radtke-Schuller S. Shamma S. A.. (2014). Emergent selectivity for task-relevant stimuli in higher-order auditory cortex. Neuron 82, 486–499. 10.1016/j.neuron.2014.02.029 24742467 PMC4048815

[B5] Atiani S. Elhilali M. David S. V. Fritz J. B. Shamma S. A. (2009). Task difficulty and performance induce diverse adaptive patterns in gain and shape of primary auditory cortical receptive fields. Neuron 61, 467–480. 10.1016/j.neuron.2008.12.027 19217382 PMC3882691

[B6] Bajo V. M. King A. J. (2010). Focusing attention on sound. Nat. Neurosci. 13, 913–914. 10.1038/nn0810-913 20661266 PMC4353843

[B7] Baluch F. Itti L. (2011). Mechanisms of top-down attention. Trends Neurosci. 34, 210–224. 10.1016/j.tins.2011.02.003 21439656

[B8] Bao S. Chan V. T. Merzenich M. M. (2001). Cortical remodelling induced by activity of ventral tegmental dopamine neurons. Nature 412, 79–83. 10.1038/35083586 11452310

[B9] Bao S. Chang E. F. Woods J. Merzenich M. M. (2004). Temporal plasticity in the primary auditory cortex induced by operant perceptual learning. Nat. Neurosci. 7, 974–981. 10.1038/nn1293 15286790

[B10] Beitel R. E. Schreiner C. E. Cheung S. W. Wang X. Merzenich M. M. (2003). Reward-dependent plasticity in the primary auditory cortex of adult monkeys trained to discriminate temporally modulated signals. Proc. Natl. Acad. Sci. U.S.A. 100, 11070–11075. 10.1073/pnas.1334187100 12941865 PMC196928

[B11] Belkin M. Niyogi P. (2004). Semi-supervised learning on Riemannian manifolds. Mach. Learn. 56, 209–239. 10.1023/B:MACH.0000033120.25363.1e

[B12] Bellur A. Elhilali M. (2015). Detection of speech tokens in noise using adaptive spectrotemporal receptive fields, in IEEE Conference on Information Sciences and Systems (CISS) (Baltimore, MD: IEEE).

[B13] Bertsekas D. (1999). Nonlinear Programming, 2nd Edn. Cambridge, MA: Athena Scientific.

[B14] Bishop C. M. (2006). Pattern Recognition and Machine Learning. Springer.

[B15] Bizley J. K. Cohen Y. E. (2013). The what, where and how of auditory-object perception. Nat. Rev. Neurosci. 14, 693–707. 10.1038/nrn3565 24052177 PMC4082027

[B16] Borji A. Itti L. (2013). State-of-the-art in visual attention modeling. IEEE Trans. Pattern Anal. Mach. Intell. 35, 185–207. 10.1109/TPAMI.2012.89 22487985

[B17] Borji A. Itti L. (2014). Optimal attentional modulation of a neural population. Front. Comput. Neurosci. 8:34. 10.3389/fncom.2014.00034 24723881 PMC3972484

[B18] Boyd S. Vandenberghe L. (2004). Convex Optimization. New York, NY: Cambridge University Press.

[B19] Brette R. Gerstner W. (2005). Adaptive exponential integrate-and-fire model as an effective description of neuronal activity. J. Neurophysiol. 94, 3637–3642. 10.1152/jn.00686.2005 16014787

[B20] Calabrese A. Schumacher J. W. Schneider D. M. Paninski L. Woolley S. M. N. (2011). A generalized linear model for estimating spectrotemporal receptive fields from responses to natural sounds. PLoS ONE 6:e16104. 10.1371/journal.pone.0016104 21264310 PMC3019175

[B21] Carlin M. A. Elhilali M. (2013). Sustained firing of central auditory neurons yields a discriminative spectro-temporal representation for natural sounds. PLoS Comput. Biol. 9:e1002982. 10.1371/journal.pcbi.1002982 23555217 PMC3610626

[B22] Carrasco M. (2011). Visual attention: the past 25 years. Vis. Res. 51, 1484–1525. 10.1016/j.visres.2011.04.012 21549742 PMC3390154

[B23] Chi T. Ru P. Shamma S. A. (2005). Multiresolution spectrotemporal analysis of complex sounds. J. Acoust. Soc. Am. 118, 887–906. 10.1121/1.1945807 16158645

[B24] David S. V. Fritz J. B. Shamma S. A. (2012). Task reward structure shapes rapid receptive field plasticity in auditory cortex. Proc. Natl. Acad. Sci. 109, 2144–2149. 10.1073/pnas.1117717109 22308415 PMC3277538

[B25] David S. V. Hayden B. Y. Mazer J. A. Gallant J. L. (2008). Attention to stimulus features shifts spectral tuning of V4 neurons during natural vision. Neuron 59, 509–521. 10.1016/j.neuron.2008.07.001 18701075 PMC2948549

[B26] Depireux D. A. Simon J. Z. Klein D. J. Shamma S. A. (2001). Spectro-temporal response field characterization with dynamic ripples in ferret primary auditory cortex. J. Neurophysiol. 85, 1220–1234. Available online at: http://jn.physiology.org/content/85/3/1220.long 11247991 10.1152/jn.2001.85.3.1220

[B27] Elhilali M. Chi T. Shamma S. A. (2003). A spectro-temporal modulation index (STMI) for assessment of speech intelligibility. Speech Commun. 41, 331–348. 10.1016/S0167-6393(02)00134-6

[B28] Elhilali M. Fritz J. B. Chi T. S. Shamma S. A. (2007). Auditory cortical receptive fields: stable entities with plastic abilities. J. Neurosci. 27, 10372–10382. 10.1523/JNEUROSCI.1462-07.2007 17898209 PMC6673154

[B29] Erkan A. Altun Y. (2010). Semi-supervised learning via generalized maximum entropy, in International Conference on Artificial Intelligence and Statistics (AISTATS), Vol. 9 (Sardinia), 209–216.

[B30] Ezzat T. Bouvrie J. V. Poggio T. (2007). Spectro-temporal analysis of speech using 2-D gabor filters, in Interspeech (Antwerp).

[B31] Feldman D. E. Brecht M. (2005). Map plasticity in somatosensory cortex. Science 310, 810–815. 10.1126/science.1115807 16272113

[B32] Frintrop S. Rome E. Christensen H. I. (2010). Computational visual attention systems and their cognitive foundations. ACM Trans. Appl. Percept. 7, 1–39. 10.1145/1658349.1658355

[B33] Fritz J. Elhilali M. Shamma S. (2005a). Active listening: task-dependent plasticity of spectrotemporal receptive fields in primary auditory cortex. Hear. Res. 206, 159–176. 10.1016/j.heares.2005.01.015 16081006

[B34] Fritz J. Elhilali M. Yin P. Harper N. Donaldson K. Shamma S. A. (2005b). Multiple auditory tasks and the single cortical neuron: salient temporal and spectral cues drive orthogonal, dynamic, task-related receptive plasticity in primary auditory cortex, in Society for Neuroscience Meeting (Washington, DC).

[B35] Fritz J. Shamma S. Elhilali M. Klein D. (2003). Rapid task-related plasticity of spectrotemporal receptive fields in primary auditory cortex. Nat. Neurosci. 6, 1216–1223. 10.1038/nn1141 14583754

[B36] Fritz J. B. David S. Shamma S. (2013). Attention and dynamic, task-related receptive field plasticity in adult auditory cortex, in Springer Handbook of Auditory Research, eds Cohen Y. E. Popper A. N. Fay R. R. (New York, NY: Springer), 251–291.

[B37] Fritz J. B. David S. V. Radtke-Schuller S. Yin P. Shamma S. A. (2010). Adaptive, behaviorally gated, persistent encoding of task-relevant auditory information in ferret frontal cortex. Nat. Neurosci. 13, 1011–1019. 10.1038/nn.2598 20622871 PMC2921886

[B38] Fritz J. B. Elhilali M. David S. V. Shamma S. A. (2007a). Auditory attention–focusing the searchlight on sound. Curr. Opin. Neurobiol. 17, 437–455. 10.1016/j.conb.2007.07.011 17714933

[B39] Fritz J. B. Elhilali M. David S. V. Shamma S. A. (2007b). Does attention play a role in dynamic receptive field adaptation to changing acoustic salience in A1? Hear. Res. 229, 186–203. 10.1016/j.heares.2007.01.009 17329048 PMC2077083

[B40] Fritz J. B. Elhilali M. Shamma S. A. (2005c). Differential dynamic plasticity of A1 receptive fields during multiple spectral tasks. J. Neurosc. 25, 7623–7635. 10.1523/JNEUROSCI.1318-05.2005 16107649 PMC6725393

[B41] Fritz J. B. Elhilali M. Shamma S. A. (2007c). Adaptive changes in cortical receptive fields induced by attention to complex sounds. J. Neurophysiol. 98, 2337–2346. 10.1152/jn.00552.2007 17699691

[B42] Gilbert C. D. Li W. (2012). Adult visual cortical plasticity. Neuron 75, 250–264. 10.1016/j.neuron.2012.06.030 22841310 PMC3408614

[B43] Grandvalet Y. Bengio Y. (2004). Semi-supervised learning by entropy minimization, in Neural Information Processing Systems (NIPS), eds Saul L. K. Weiss Y. Bottou L. (Vancouver, BC).

[B44] Grant M. Boyd S. (2008). Graph implementations for nonsmooth convex programs, in Recent Advances in Learning and Control, Lecture Notes in Control and Information Sciences, eds Blondel V. Boyd S. Kimura H. (Berlin: Springer-Verlag Limited), 95–110.

[B45] Grant M. Boyd S. (2014). CVX: Matlab Software for Disciplined Convex Programming, Version 2.1. Availabe online at: http://cvxr.com/cvx

[B46] Griffiths T. D. Warren J. D. (2004). What is an auditory object? Nat. Rev. Neurosci. 5, 887–892. 10.1038/nrn1538 15496866

[B47] Itti L. Koch C. (2001). Computational modelling of visual attention. Nat. Rev. Neurosci. 2, 194–203. 10.1038/35058500 11256080

[B48] Itti L. Rees G. Tsotsos J. K. (eds.) (2005). Neurobiology of Attention. Waltham, MA: Academic Press.

[B49] Kilgard M. P. Merzenich M. M. (1998). Plasticity of temporal information processing in the primary auditory cortex. Nat. Neurosci. 1, 727–731. 10.1038/3729 10196590 PMC2948964

[B50] Kilgard M. P. Pandya P. K. Vazquez J. Gehi A. Schreiner C. E. Merzenich M. M. (2001). Sensory input directs spatial and temporal plasticity in primary auditory cortex. J. Neurophysiol. 86, 326–338. 11431514 10.1152/jn.2001.86.1.326

[B51] Klein D. J. Simon J. Z. Depireux D. A. Shamma S. A. (2006). Stimulus-invariant processing and spectrotemporal reverse correlation in primary auditory cortex. J. Comput. Neurosci. 20, 111–136. 10.1007/s10827-005-3589-4 16518572

[B52] Krumbholz K. Eickhoff S. B. Fink G. R. (2007). Feature- and object-based attentional modulation in the human auditory “where” pathway. J. Cogn. Neurosci. 19, 1721–1733. 10.1162/jocn.2007.19.10.1721 18271742

[B53] Mandairon N. Linster C. (2009). Odor perception and olfactory bulb plasticity in adult mammals. J. Neurophysiol. 101, 2204–2209. 10.1152/jn.00076.2009 19261715

[B54] Martínez-Trujillo J. Treue S. (2002). Attentional modulation strength in cortical area MT depends on stimulus contrast. Neuron 35, 365–370. 10.1016/S0896-6273(02)00778-X 12160753

[B55] Mesgarani N. Chang E. F. (2012). Selective cortical representation of attended speaker in multi-talker speech perception. Nature 485, 233–236. 10.1038/nature11020 22522927 PMC3870007

[B56] Mesgarani N. David S. V. Fritz J. B. Shamma S. A. (2014). Mechanisms of noise robust representation of speech in primary auditory cortex. Proc. Natl. Acad. Sci. U.S.A. 111, 6792–6797. 10.1073/pnas.1318017111 24753585 PMC4020083

[B57] Mesgarani N. Fritz J. Shamma S. (2010). A computational model of rapid task-related plasticity of auditory cortical receptive fields. J. Comput. Neurosci. 28, 19–27. 10.1007/s10827-009-0181-3 19711179 PMC3973422

[B58] Miller E. K. Buschman T. J. (2013). Cortical circuits for the control of attention. Curr. Opin. Neurobiol. 23, 216–222. 10.1016/j.conb.2012.11.011 23265963 PMC3709832

[B59] Motter B. C. (1993). Focal attention produces spatially selective processing in visual cortical areas V1, V2, and V4 in the presence of competing stimuli. J. Neurophysiol. 70, 909–919. 8229178 10.1152/jn.1993.70.3.909

[B60] Navalpakkam V. Itti L. (2007). Search goal tunes visual features optimally. Neuron 53, 605–617. 10.1016/j.neuron.2007.01.018 17296560

[B61] Olshausen B. A. Anderson C. H. Van Essen D. C. (1993). A neurobiological model of visual attention and invariant pattern recognition based on dynamic routing of information. J. Neurosci. 13, 4700–4719. 8229193 10.1523/JNEUROSCI.13-11-04700.1993 PMC6576339

[B62] Ostojic S. Brunel N. (2011). From spiking neuron models to linear-nonlinear models. PLoS Comput. Biol. 7:e1001056. 10.1371/journal.pcbi.1001056 21283777 PMC3024256

[B63] Paninski L. (2004). Maximum likelihood estimation of cascade point-process neural encoding models. Netw. Comput. Neural Syst. 15, 243–262. 10.1088/0954-898X/15/4/002 15600233

[B64] Patil K. Pressnitzer D. Shamma S. Elhilali M. (2012). Music in our ears: the biological bases of musical timbre perception. PLoS Comput. Biol. 8:e1002759. 10.1371/journal.pcbi.1002759 23133363 PMC3486808

[B65] Rasmusson D. D. Smith S. A. Semba K. (2007). Inactivation of prefrontal cortex abolishes cortical acetylcholine release evoked by sensory or sensory pathway stimulation in the rat. Neuroscience 149, 232–241. 10.1016/j.neuroscience.2007.06.057 17850979

[B66] Reynolds J. H. Heeger D. J. (2009). The normalization model of attention. Neuron 61, 168–185. 10.1016/j.neuron.2009.01.002 19186161 PMC2752446

[B67] Schreiner C. E. Polley D. B. (2014). Auditory map plasticity: diversity in causes and consequences. Curr. Opin. Neurobiol. 24, 143–156. 10.1016/j.conb.2013.11.009 24492090 PMC4206409

[B68] Shamma S. Fritz J. David S. Winkowski D. Yin P. Elhilali M. (2010). Correlates of auditory attention and task performance in primary auditory and prefrontal cortex, in The Neurophysiological Bases of Auditory Perception, eds Lopez-Poveda E. Palmer A. Meddis R. (New York, NY: Springer), 555–570.

[B69] Shinn-Cunningham B. G. (2008). Object-based auditory and visual attention. Trends Cogn. Sci. 12, 182–186. 10.1016/j.tics.2008.02.003 18396091 PMC2699558

[B70] Shuai L. Elhilali M. (2014). Task-dependent neural representations of salient events in dynamic auditory scenes. Front. Neurosci. 8:203. 10.3389/fnins.2014.00203 25100934 PMC4104552

[B71] Spitzer H. Desimone R. Moran J. (1988). Increased attention enhances both behavioral and neuronal performance. Science 240, 338–340. 10.1126/science.3353728 3353728

[B72] Theunissen F. E. Sen K. Doupe A. J. (2000). Spectral-temporal receptive fields of nonlinear auditory neurons obtained using natural sounds. J. Neurosci. 20, 2315–2331. Available online at: http://www.jneurosci.org/content/20/6/2315.full 10704507 10.1523/JNEUROSCI.20-06-02315.2000 PMC6772498

[B73] Treisman A. (1996). The binding problem. Curr. Opin. Neurobiol. 6, 171–178. 10.1016/S0959-4388(96)80070-5 8725958

[B74] Treue S. Martínez Trujillo J. C. (1999). Feature-based attention influences motion processing gain in macaque visual cortex. Nature 399, 575–579. 10.1038/21176 10376597

[B75] Truccolo W. Eden U. T. Fellows M. R. Donoghue J. P. Brown E. N. (2005). A point process framework for relating neural spiking activity to spiking history, neural ensemble, and extrinsic covariate effects. J. Neurophysiol. 93, 1074–1089. 10.1152/jn.00697.2004 15356183

[B76] Tsotsos J. Culhane S. Wai W. K. Lai Y. (1995). Modeling visual attention via selective tuning. Artif. Intell. 78, 507–545. 10.1016/0004-3702(95)00025-9

[B77] Womelsdorf T. Anton-Erxleben K. Pieper F. Treue S. (2006). Dynamic shifts of visual receptive fields in cortical area MT by spatial attention. Nat. Neurosci. 9, 1156–1160. 10.1038/nn1748 16906153

[B78] Yin P. Fritz J. B. Shamma S. A. (2014). Rapid spectrotemporal plasticity in primary auditory cortex during behavior. J. Neurosci. 34, 4396–4408. 10.1523/JNEUROSCI.2799-13.2014 24647959 PMC3960477

