# Supplementary material for: Modeling attention-driven plasticity in auditory cortical receptive fields
Source: Front Comput Neurosci. 2015 Aug 19;9:106. doi: 10.3389/fncom.2015.00106 (PMC4541291; doi:10.3389/fncom.2015.00106)
Supplement: Supplementary file 1 [file Presentation1.PDF]

Frontiers Computational Neuroscience

Research Article
2 December 2014

Supplementary Material: Modeling attention-driven plasticity in auditory cortical receptive fields

Michael A. Carlin and Mounya Elhilali *

Laboratory for Computational Audio Perception, Department of Electrical and Computer Engineering, Johns Hopkins University, Baltimore, MD, USA

Correspondence*:
Mounya Elhilali
Department of Electrical and Computer Engineering, Johns Hopkins University,
3400 North Charles Street, Baltimore, MD, 21218, USA, mounya@jhu.edu

SUPPLEMENTARY FIGURE 1

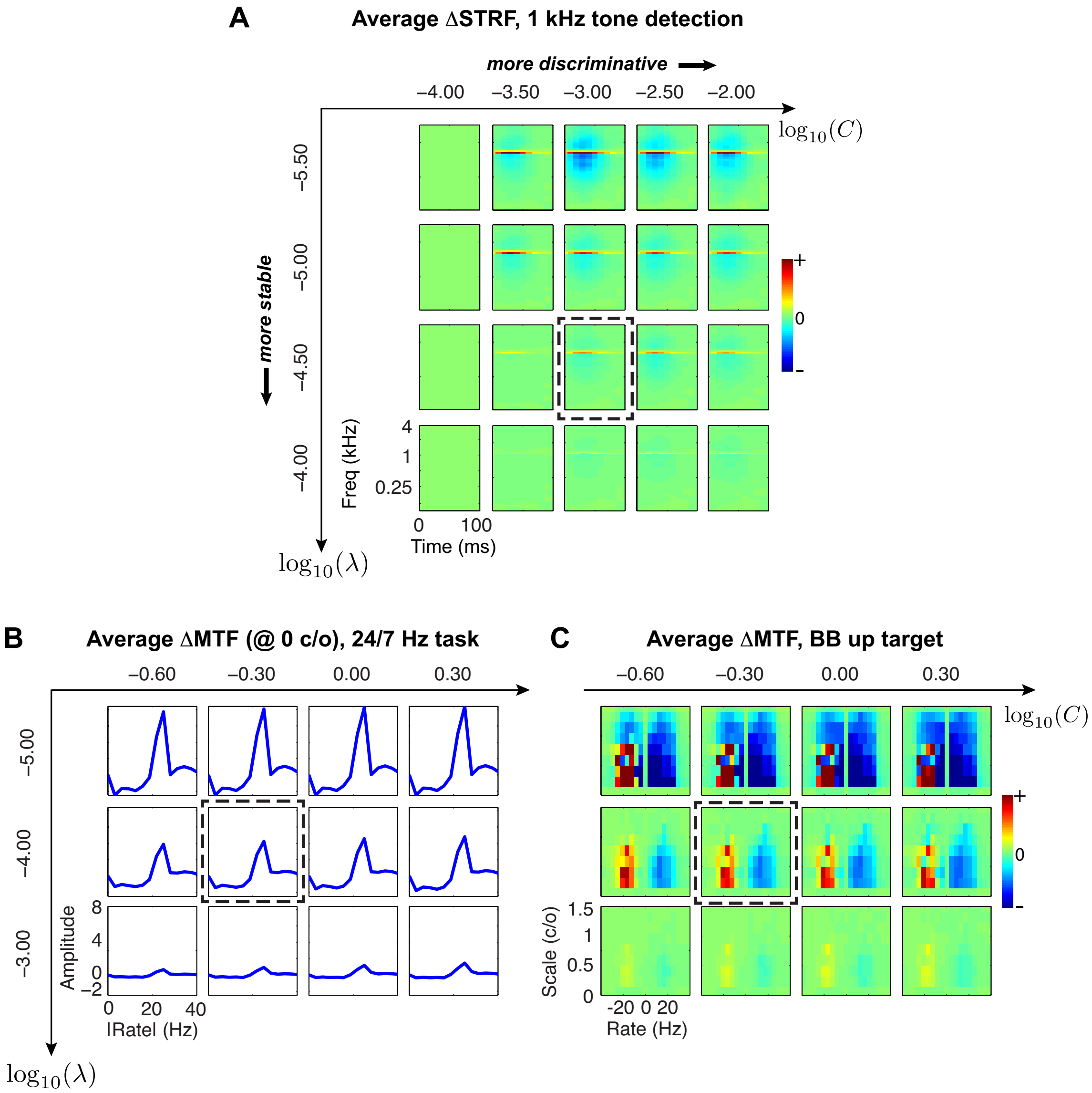

Supplementary Figure 1. Influence of model hyperparameters on population plasticity patterns. *Feature-Based Model, tone detection:* Panel A shows the average $\Delta STRF$ for the 1 kHz tone detection task as a function of model hyperparameters C and λ. Increasing C emphasizes discriminability in the overall objective function (Eq. 4 in the main text) whereas increasing λ emphasizes stability. Similar patterns were observed for the other tasks considered for the Feature-Based Model. *Object-Based Model, click rate discrimination:* Panel B shows the average ΔMTF at 0 c/o, folded along the scale axis for clarity. Changes to C and λ vary the magnitude of the plasticity effect as with the Feature-Based Model. Similar patterns were observed for the other tasks. *Object-Based Model, spectrotemporal modulation noise discrimination:* Finally, panel C shows the average ΔMTF for the *BB Up* target. Again, the size of the plasticity effect depends on choice of hyperparameters. Similar patterns were observed for the other tasks. For all panels, dashed boxes indicate the hyperparameters used for simulation and analysis for the various tasks described in the text.

SUPPLEMENTARY TEXT 1

INCLUDING A SPECTRO-TEMPORAL MASK IN THE OBJECT-BASED MODEL

As in the Feature-Based formulation, including a spectro-temporal mask in the Object-Based Model is desirable to guarantee locality of STRF adaptations. For technical reasons, however, use of a mask in the current formulation yields optimal STRF updates that fail to strictly satisfy the contrast filtering hypothesis, i.e., enhancement of target features and suppression of non-target features. As we show below, inclusion of the mask still induces adaptations that improve discrimination (by definition of the objective function), but it remains unclear whether this formulation of the model is suitable for accounting for physiological results. In this section, we elaborate on the details.

To begin, we model neural firing rate with the inclusion of a mask as

$$r_k(t,f;m) = [m_k(t,f)\cdot h_k^A(t,f)] *_{tf} s_m(t,f)$$

with Fourier domain representation

$$R_k(\omega,\Omega;m) = [M_k(\omega,\Omega) *_{\omega\Omega} H_k^A(\omega,\Omega)]\cdot S_m(\omega,\Omega)$$

where $M_k(\omega,\Omega), H_k(\omega,\Omega), S_m(\omega,\Omega)$ are the 2D Discrete Fourier Transforms of the mask, STRF, and stimulus, respectively. Expanding this term yields

$$\begin{aligned} R_k(\omega,\Omega;m) &= \sum_{ln} |M_k(\omega-l,\Omega-n)|\cdot|H_k^A(l,n)|\cdot|S_m(l,n)|\cdot\exp\{j\phi_{ln}(k,m)\} \\ &= \sum_{ln} |M_k(\omega-l,\Omega-n)|\cdot|H_k^A(l,n)|\cdot|S_m(l,n)|\cdot(\cos\phi_{ln}(k,m)+j\sin\phi_{ln}(k,m)) \\ &= \mathfrak{Re}\{R_k(\omega,\Omega;m)\} + j\mathfrak{Im}\{R_k(\omega,\Omega;m)\} \end{aligned}$$

where

$$\begin{aligned} \phi_{ln}(k,m) &= \angle M_k(\omega-l,\Omega-n) + \angle H_k^0(l,n) + \angle S_m(l,n) \\ \mathfrak{Re}\{R_k(\omega,\Omega;m)\} &= \sum_{ln} |M_k(\omega-l,\Omega-n)|\cdot|H_k^A(l,n)|\cdot|S_m(l,n)|\cdot\cos\phi_{ln}(k,m) \\ \mathfrak{Im}\{R_k(\omega,\Omega;m)\} &= \sum_{ln} |M_k(\omega-l,\Omega-n)|\cdot|H_k^A(l,n)|\cdot|S_m(l,n)|\cdot\sin\phi_{ln}(k,m) \end{aligned}$$

Note that because we optimize only the modulation profiles of the STRFs, we keep the phase of the STRFs fixed to those of the initial filters. The power spectrum of the neural response can therefore be expressed as

$$|R_k(\omega,\Omega;m)|^2 = \mathfrak{Re}^2\{R_k(\omega,\Omega;m)\} + \mathfrak{Im}^2\{R_k(\omega,\Omega;k)\}$$

We use the power spectrum in the objective function for mathematical convenience when computing gradients.

Next, consider the objective function defined as

$$J(\mathbf{w},\widehat{\mathcal{H}}_A) := \frac{1}{2}||\mathbf{w}||_2^2 - C\cdot\left\langle \log\sigma\left(y_m\left(w_0+\sum_k w_k\sum_{\omega\Omega}|R_k(\omega,\Omega;m)|^2\right)\right)\right\rangle_m + \frac{\lambda}{2}\sum_k||\Delta_k(\omega,\Omega)||_F^2$$

where $\widehat{\mathcal{H}}_A := \{|H_k(\omega,\Omega)|\}_{k=1}^K$ is the collection of ensemble modulation profiles and $\Delta_k(\omega,\Omega) := |H_k^0(\omega,\Omega)| - |H_k^A(\omega,\Omega)|$. As before, the optimal STRF modulation profiles are achieved by searching

for a minimum of the of the objective function, i.e., when $\nabla_{|H_k^A(\omega,\Omega)|} J = 0$. Assuming this minimum occurs in the feasible set formed by the nonnegativity constraints on the regressor and STRF modulation profiles, we find

$$|H_k^A(\omega', \Omega')| = |H_k^0(\omega', \Omega')| + \frac{C}{\lambda} \cdot \left\langle y_m [1 - \sigma(y_m \mathbf{w}^T \mathbf{R}_m)] \cdot \frac{\partial |R_k(\omega, \Omega)|^2}{\partial |H_k^A(\omega', \Omega')|} \right\rangle_m$$

where

$$\frac{\partial |R_k(\omega, \Omega)|^2}{\partial |H_k^A(\omega', \Omega')|} = 2 \cdot \mathfrak{Re}\{R_k(\omega, \Omega; m)\} \cdot \big[|M_k(\omega - \omega', \Omega - \Omega')| \cdot |S_m(\omega', \Omega')| \cdot \cos \phi_{\omega'\Omega'}(k, m)\big] + 2 \cdot \mathfrak{Im}\{R_k(\omega, \Omega; m)\} \cdot \big[|M_k(\omega - \omega', \Omega - \Omega')| \cdot |S_m(\omega', \Omega')| \cdot \sin \phi_{\omega'\Omega'}(k, m)\big]$$

for some particular (ω', Ω'). Recall that in the original Object-Based Model formulation, modulations associated with target stimuli were guaranteed to be enhanced whereas modulations associated with non-target stimuli were guaranteed to be suppressed. This was due to the nonnegativity of the filter response gradient $\partial |R_k(\omega, \Omega)|^2 / \partial |H_k^A(\omega', \Omega')|$, and we therefore concluded that this model was strictly consistent with the contrast filtering hypothesis. However, observe that when the model includes a spectro-temporal mask, the filter response gradient involves $\sin$ and $\cos$ terms that necessarily take values in $[-1, +1]$. As a consequence, the gradient is not necessarily nonnegative and we are *no longer guaranteed that target modulations are enhanced and non-target modulations suppressed*. For this reason, this formulation of the model is *not* consistent with the contrast filtering hypothesis.

Under what conditions is the nonlinear formulation with a mask consistent with the contrast filtering hypothesis? Observe that when $0 \leq \phi_{\omega'\Omega'}(k, m) < \pi/2$, the $\sin$ and $\cos$ terms are positive and consequently the filter response gradient is also positive. However, unless we optimize the full Fourier domain representation, which gives access to the STRF phase but defeats the purpose of optimizing the modulation profiles of the STRFs, we have no control over whether this inequality is satisfied.
